# A Shuttle-Vector System Allows Heterologous Gene Expression in the Thermophilic Methanogen Methanothermobacter thermautotrophicus ΔH

**DOI:** 10.1128/mBio.02766-21

**Published:** 2021-11-23

**Authors:** Christian Fink, Sebastian Beblawy, Andreas M. Enkerlin, Lucas Mühling, Largus T. Angenent, Bastian Molitor

**Affiliations:** a Environmental Biotechnology Group, University of Tübingen, Tübingen, Germany; b Cluster of Excellence – Controlling Microbes to Fight Infections, University of Tübingen, Tübingen, Germany; c Max Planck Fellow, Max Planck Institute for Developmental Biology, Tübingen, Germany; University of Vienna

**Keywords:** *Archaea*, genetics, *Methanothermobacter*, shuttle vector, β-galactosidase, formate, beta-galactosidase

## Abstract

Thermophilic *Methanothermobacter* spp. are used as model microbes to study the physiology and biochemistry of the conversion of molecular hydrogen and carbon dioxide into methane (i.e., hydrogenotrophic methanogenesis). Yet, a genetic system for these model microbes was missing despite intensive work for four decades. Here, we report the successful implementation of genetic tools for Methanothermobacter thermautotrophicus ΔH. We developed shuttle vectors that replicated in Escherichia coli and *M. thermautotrophicus* ΔH. For *M. thermautotrophicus* ΔH, a thermostable neomycin resistance cassette served as the selectable marker for positive selection with neomycin, and the cryptic plasmid pME2001 from Methanothermobacter marburgensis served as the replicon. The shuttle-vector DNA was transferred from E. coli into *M. thermautotrophicus* ΔH via interdomain conjugation. After the successful validation of DNA transfer and positive selection in *M. thermautotrophicus* ΔH, we demonstrated heterologous gene expression of a thermostable β-galactosidase-encoding gene (*bgaB*) from Geobacillus stearothermophilus under the expression control of four distinct synthetic and native promoters. In quantitative *in-vitro* enzyme activity assay, we found significantly different β-galactosidase activity with these distinct promoters. With a formate dehydrogenase operon-encoding shuttle vector, we allowed growth of *M. thermautotrophicus* ΔH on formate as the sole growth substrate, while this was not possible for the empty-vector control.

## INTRODUCTION

Methanogenesis is the biological production of methane, which is catalyzed by methane-producing archaea (methanogens). Hydrogenotrophic methanogens grow with molecular hydrogen (electron donor) and carbon dioxide (electron acceptor and carbon source) as the substrates (1). The *Methanobacteriales* species Methanothermobacter thermautotrophicus and Methanothermobacter marburgensis have been studied as model microbes for the biochemistry of hydrogenotrophic methanogenesis, and deep insights into their energy and carbon metabolism have been acquired (1–3). For example, comparative genome analyses of *M. thermautotrophicus* and *M. marburgensis* revealed the genes that are most likely required for hydrogenotrophic methanogenesis (4), and a plethora of studies unraveled the mechanism of key enzymes such as the methyl-coenzyme M reductase (reviewed in reference 5). Furthermore, the pseudomurein-containing cell wall, which is specific to *Methanobacteriales*, has attracted research on *Methanothermobacter* spp. (6, 7).

Additionally, *Methanothermobacter* spp. have been implemented as biocatalysts in the power-to-gas platform on a large scale already (8, 9), because high cell densities and high methane production rates can be achieved (10, 11). In the power-to-gas platform, molecular hydrogen from the electrolysis of water with surplus electric power from renewable resources is combined with carbon dioxide, and these gases are converted into methane (8). This methane (i.e., renewable natural gas) can be introduced into the natural gas grid with large storage capacities to substitute for fossil natural gas. With genetic engineering of *Methanothermobacter* spp., the metabolism of the biocatalysts can, for example, be improved to maximize methane production rates or be amended for an expanded substrate or product spectrum, which would allow conversion of the power-to-gas platform into a broader power-to-chemical (i.e., power-to-x) platform (9).

This long-lasting interest in the biochemistry, physiology, and application of *Methanothermobacter* spp. led to extensive attempts to establish genetic tools in the past (12–14). Successes were reported in *M. marburgensis* for reversing spontaneous amino acid-auxotrophic phenotypes via generalized transduction from the wild-type strain with the virus ΨM2 (15) or via the uptake of free DNA (i.e., natural competence) with high-molecular-weight genomic DNA (16–18). In addition, 5-fluorouracil-resistant phenotypes were reported to be conferred with high-molecular-weight genomic DNA from a spontaneously resistant strain via natural competence in *M. marburgensis* with a higher frequency than the rate for the occurrence of spontaneous resistance (16). Other spontaneously antibiotic-resistant *Methanothermobacter* strains were investigated as potential sources for genes that can be used as selectable markers, including pseudomonic acid (17)- and neomycin (19)-resistant strains. Furthermore, several potential Escherichia coli-*Methanothermobacter* shuttle vectors have been constructed and replicated in E. coli, which were based on the cryptic plasmid (i.e., no physiological function has been assigned to the plasmid) pME2001 from *M. marburgensis* (12). Nevertheless, until now none of these approaches was translated into a reliable genetic system for *Methanothermobacter* spp.

Spurred by the recurring interest in *Methanothermobacter* spp. as model microbes for methanogenic biochemistry and physiology with a long history of fundamental research, and as biotechnologically relevant microbes in power-to-gas processes, we set out to utilize modern molecular biology tools and the knowledge of genetic tools for other methanogens and thermophilic microbes to develop a genetic system for *M. thermautotrophicus* ΔH. We report the successful establishment of genetic tools for *M. thermautotrophicus* ΔH, which now provide the basis to investigate hypotheses from four decades of research on the evolution, physiology, and biochemistry of *Methanothermobacter* spp. on a genetic level.

## RESULTS

### Clonal populations of *M. thermautotrophicus* ΔH can be obtained on solidified medium plates as individual colonies with high plating efficiencies.

The first requirement to allow genetic work with any given microbe is the capability to isolate clonal populations. This is typically achieved by plating microbial cultures on solidified medium plates and by selecting individual colonies. Therefore, we first reproduced the high plating efficiencies that have been reported in the literature for *Methanothermobacter* spp. (20). We investigated three common plating techniques (spot, spread, and pour plating) and compared factors that influenced plating efficiency (i.e., individual colonies per cell count of plated microbial cells [Materials and Methods]). We achieved dense growth with spot plating, but individual colonies were barely distinguishable with this plating technique, while we achieved plating efficiencies between 1 and 5% with spread plating, and 50% and higher with pour plating (see Text S1A and Fig. S1 in the supplemental material).

10.1128/mBio.02766-21.1 TEXT S1 Supplemental results (A to I) and discussion (J). (A) Differences in plating efficiencies for various plating techniques. (B) Description of growth-inhibiting effects of antibiotics. (C) Details on the design of the modular shuttle-vector system pMVS. (D) Analysis of successful DNA transfer via site-specific PCR. (E) Retransformation of E. coli with plasmid extracts from *M. thermautotrophicus* ΔH. (F) Approximation of the conjugation frequency. (G) Segregational stability of shuttle vector under nonselective conditions. (H) Control experiments for conjugational DNA transfer. (I) Quantitative β-galactosidase enzyme activity assay. (J) Discussion on plating efficiency of *M. thermautotrophicus* ΔH. Download Text S1, DOCX file, 0.06 MB. Copyright © 2021 Fink et al. 2021 Fink et al. https://creativecommons.org/licenses/by/4.0/ This content is distributed under the terms of the Creative Commons Attribution 4.0 International license.

10.1128/mBio.02766-21.2 FIG S1 (A) Spot plating of *M. thermautotrophicus* ΔH cells on solidified medium plates. (B) Pour plating of 1 × 10^4^ *M. thermautotrophicus* ΔH cells on solidified medium plates. (C) Spread plating of 1 × 10^4^ *M. thermautotrophicus* ΔH cells on solidified medium plates. (D) Comparison of the influence from various factors on the plating efficiency with spread plating of 1 × 10^4^ *M. thermautotrophicus* ΔH cells and pour plating of 1 × 10^3^ *M. thermautotrophicus* ΔH cells, including the influence of paper clips, 0.1 vol% hydrogen sulfide (H_2_S) in the headspace gas mixture, exponential growth phase (Exp), and stationary growth phase (Stat). The bars give the average percentage (log scale) of individual colonies per cell count of initial microbial cells used for plating from technical replicates (*n *= 3; *n *= 4 for spread plating with paper clips and H_2_S in exponential growth phase) with error bars indicating standard deviation. (E) Influence of paper clips on the lid elevation. The arrow demonstrates the increase of lid/plate space compared for a plate without paper clips (upper picture) and one with paper clips (lower picture). Download FIG S1, TIF file, 0.3 MB. Copyright © 2021 Fink et al. 2021 Fink et al. https://creativecommons.org/licenses/by/4.0/ This content is distributed under the terms of the Creative Commons Attribution 4.0 International license.

### *M. thermautotrophicus* ΔH is sensitive to antibiotics commonly used in methanogen genetic systems.

To find a suitable selection pressure for the positive selection of genetically modified cells, we analyzed several antibiotics such as simvastatin and neomycin (Text S1B). For both antibiotics, thermostable selectable markers are available, which have been successfully used in thermophilic nonmethanogenic microbes, such as Pyrococcus furiosus and Thermococcus kodakarensis (simvastatin) (21, 22), as well as *Thermoanaerobacter* spp. (neomycin) (23), but recently also in the thermophilic methanogens Methanocaldococcus jannaschii (simvastatin) and Methanoculleus thermophilus (neomycin) (24, 25). Both simvastatin and neomycin efficiently inhibited growth of *M. thermautotrophicus* ΔH cells in liquid culture at concentrations of 21.5 μg/ml and 250 μg/ml, respectively (Text S1B and Fig. S2 and S3). While these antibiotics inhibited growth of *M. thermautotrophicus* ΔH, we observed the appearance of spontaneously resistant *M. thermautotrophicus* ΔH cells for both antibiotics (Text S1B and Fig. S2 and S3). The incubation period for the appearance of spontaneously resistant *M. thermautotrophicus* ΔH cells differed for each antibiotic compared to the incubation period for nonselective growth of wild-type cells (16 to 24 h). We observed inhibition of growth for at least 48 h with simvastatin and 60 h with neomycin at the concentrations indicated above (Text S1B). Because we found that neomycin inhibits growth of *M. thermautotrophicus* ΔH for a longer incubation period than that for simvastatin, we decided to focus on neomycin as the selection pressure to develop the first selectable marker for *M. thermautotrophicus* ΔH.

10.1128/mBio.02766-21.3 FIG S2 Measurement of OD_600_ after different incubation periods at 60°C (0 h, 24 h, 48 h, and 60 h) with initially 5 × 10^5^ cells/ml of wild-type *M. thermautotrophicus* ΔH in liquid mineral medium containing neomycin concentrations of 0 μg/ml (long-dashed line, *n *= 1), 50 μg/ml (dotted line), 100 μg/ml (short-dashed line), and 250 μg/ml (black solid line). For comparison to wild-type *M. thermautotrophicus* ΔH, initially 5 × 10^5^ cells/ml of pMVS-V1-carrying *M. thermautotrophicus* ΔH were incubated in selective liquid mineral medium with 250 μg/ml neomycin (gray solid line, *n *= 1) and analyzed after the same incubation periods. Average (*n *= 3) with error bars indicating standard deviation. Download FIG S2, EPS file, 1.3 MB. Copyright © 2021 Fink et al. 2021 Fink et al. https://creativecommons.org/licenses/by/4.0/ This content is distributed under the terms of the Creative Commons Attribution 4.0 International license.

10.1128/mBio.02766-21.4 FIG S3 (A) Individual colonies from 1 × 10^8^ wild-type *M. thermautotrophicus* ΔH cells (gray bars), which were pour plated on solidified medium plates containing simvastatin concentrations ranging from 13 μg/ml to 21.5 μg/ml. (B) Individual colonies from 1 × 10^3^ wild-type *M. thermautotrophicus* ΔH cells (gray bars) and pMVS-V1-carrying *M. thermautotrophicus* ΔH (black bars, *n *= 1), which were pour plated on solidified medium plates containing neomycin concentrations ranging from 0 μg/ml to 250 μg/ml. Average (*n *= 3) with error bars indicating standard deviation for wild-type *M. thermautotrophicus* ΔH. Download FIG S3, EPS file, 1.3 MB. Copyright © 2021 Fink et al. 2021 Fink et al. https://creativecommons.org/licenses/by/4.0/ This content is distributed under the terms of the Creative Commons Attribution 4.0 International license.

### A modular plasmid design enables the fast generation of shuttle vectors to test genetic elements for functionality in *M. thermautotrophicus* ΔH.

Before we focused on the neomycin-selectable marker, we had constructed a subset of plasmids that would allow us to test various approaches for the transfer of DNA into *M. thermautotrophicus* ΔH and for positive selection (Materials and Methods). To ease exchangeability of genetic elements in shuttle vectors, and to allow fast adaptation to new findings, we decided on a modular plasmid design. Inspired by the pSEVA system for Gram-negative bacteria (26), and the pMTL80000 system for *Clostridia* (27), we established the *Methanothermobacter* vector system (pMVS) design.

**FIG 1:**
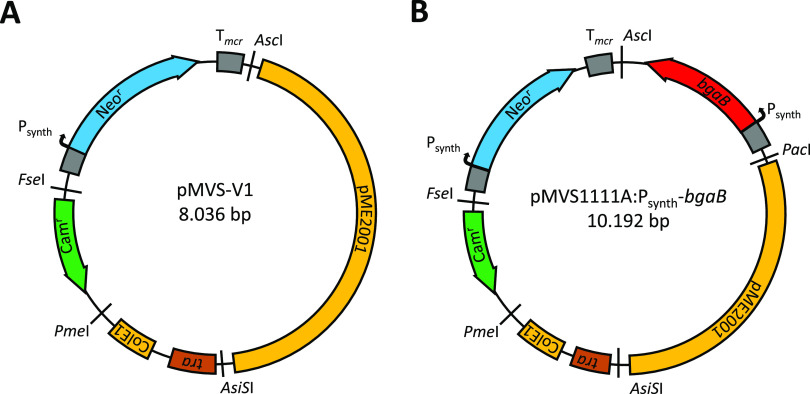
Plasmid maps of the *Methanothermobacter* vector system (pMVS). (A) pMVS-V1 consists of four modules, which are intersected by the 8-bp restriction enzyme recognition sites AscI, FseI, PmeI, and AsiSI. The four modules are the replicon for E. coli (ColE1, *tra*), the selectable marker for E. coli (Cam^r^), the replicon for *M. thermautotrophicus* ΔH (pME2001), and the selectable marker for *M. thermautotrophicus* ΔH (Neo^r^). (B) pMVS1111A:P_synth_-*bgaB* consists of five modules, which are intersected by the 8-bp restriction enzyme recognition sites AscI, FseI, PmeI, AsiSI, and PacI. The five modules are as in pMVS-V1 with the following differences: the replicon for *M. thermautotrophicus* ΔH is flanked by AsiSI and PacI, and the shuttle vector contains the *bgaB* gene in the application module, which is flanked by PacI and AscI. P_synth_, synthetic promoter sequence, which is based on P*_hmtB_*; T*_mcr_*, terminator sequence from the *mcr* operon of M. voltae. The nomenclature for the pMVS design is realized by adding a four-digit code after pMVS for the definition of the first four modules, which defines the plasmid backbone with basic functions for replication and selection in E. coli (modules 1 and 2) and *M. thermautotrophicus* ΔH (modules 3 and 4). Additional large capital letters can be amended to each digit to define differences, such as varying promoter sequences, in a given module. For the fifth (application) module, a descriptive name is added after the four-digit code, which allows staying as flexible as possible with the genetic cargo used (without a limitation to nine digits), while staying within the pMVS design boundaries.

The pMVS design consists of five modules, which are separated by rare 8-bp recognition sequences for the restriction enzymes PmeI, AsiSI, FseI, AscI, and PacI (Fig. 1). To follow the pMVS design, these rare restriction enzyme-recognition sequences need to stay unique to grant exchangeability of the modules by restriction/ligation cloning. The five modules are (restriction enzyme boundaries are given in parentheses) (i) the replicon for E. coli (AsiSI, PmeI), (ii) the selectable marker for E. coli (PmeI, FseI), (iii) the replicon for *M. thermautotrophicus* ΔH (AsiSI, PacI), (iv) the selectable marker for *M. thermautotrophicus* ΔH (AscI, FseI), and (v) an application module that can be used to include any genetic cargo such as a reporter gene or another gene of interest (PacI, AscI) (Fig. 1 and Text S1C).

Based on the first shuttle vector (pMVS-V1) that led to successful DNA transfer and selection protocols, as described below, we defined pMVS-V1 as our archetype shuttle vector (Fig. 1A), with a combination of (i) the ColE1-derived replicon for E. coli in combination with the conjugational transfer function (*tra* region) from RK2 (27), (ii) the chloramphenicol-selectable marker (Cam^r^) for E. coli (27), (iii) the entire cryptic plasmid pME2001 from *M. marburgensis* as the replicon for *M. thermautotrophicus* ΔH (14), and (iv) the thermostable neomycin-selectable marker (Neo^r^) (28) for *M. thermautotrophicus* ΔH under the control of the P_synth_ promoter sequence (22), but without an application module and a PacI recognition sequence (Fig. 1A). After we had demonstrated the functionality of pMVS-V1, our first complete shuttle vector, pMVS1111A-P_synth_-*bgaB*, was then constructed based on this archetype shuttle vector. pMVS1111A-P_synth_-*bgaB* contains the β-galactosidase-encoding gene *bgaB* (see below) and an additional PacI site, which completes the application module (Fig. 1B, Materials and Methods, and Text S1C).

### DNA transfer into *M. thermautotrophicus* ΔH is possible via interdomain conjugation with E. coli S17-1.

**FIG 2:**
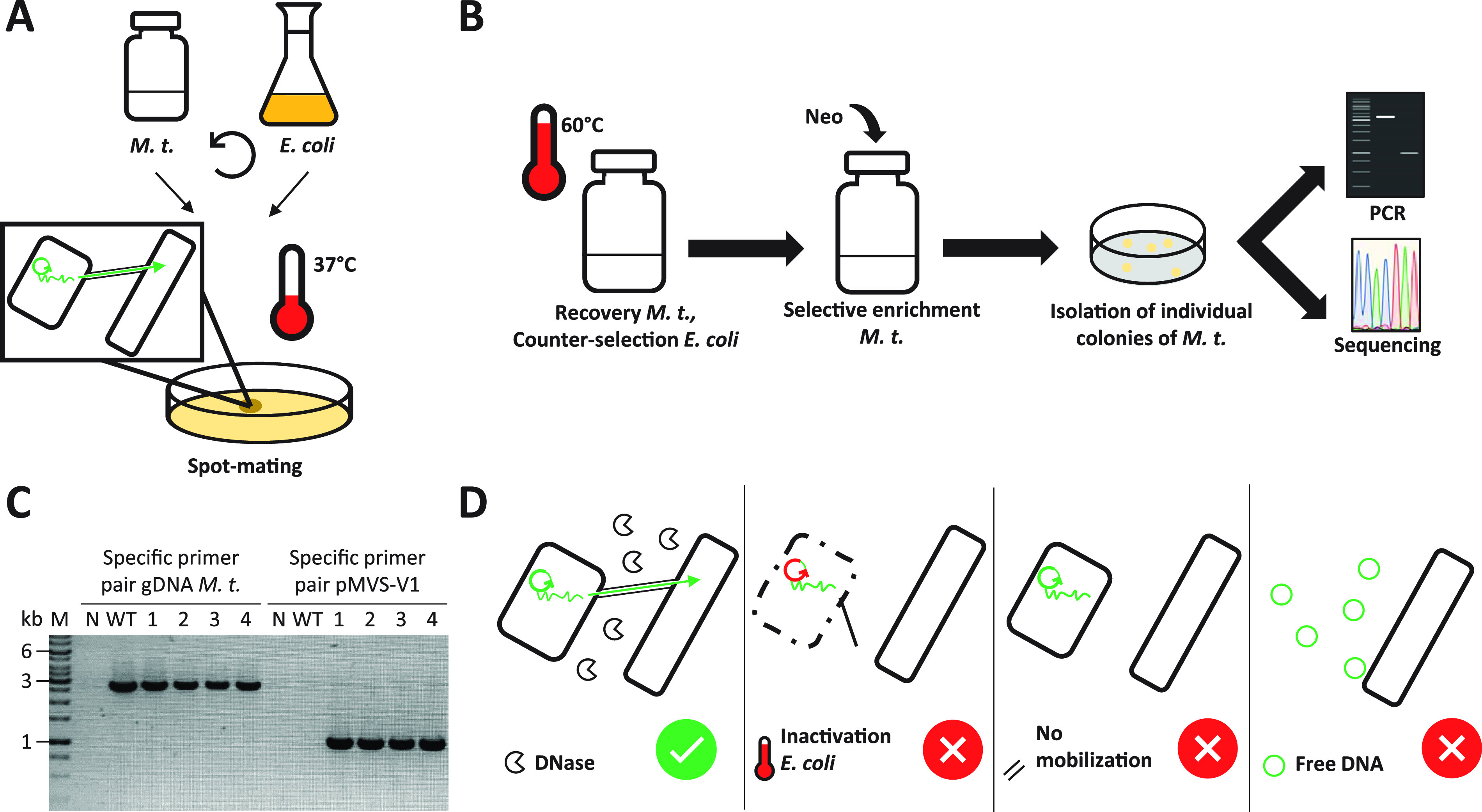
Schematic depiction and analysis of interdomain conjugation between E. coli S17-1 and *M. thermautotrophicus* ΔH. (A) Wild-type *M. thermautotrophicus* ΔH (*M. t*.) and the shuttle vector-carrying E. coli were harvested by centrifugation, mixed, and spotted on solidified medium plates that support growth of both microbes. During the spot-mating step at 37°C, the DNA transfer process via conjugation takes place (small scheme). (B) The process to isolate and identify individual colonies of genetically modified *M. thermautotrophicus* ΔH in the standard protocol. After the spot-mating step, *M. thermautotrophicus* ΔH cells were recovered in nonselective liquid mineral medium at 60°C, and afterward, transconjugants were enriched in neomycin (Neo)-containing selective liquid mineral medium at 60°C. Individual colonies were obtained from plating the enrichment culture. Those colonies were analyzed by PCR and Sanger sequencing. (C) PCR analysis of four respective transconjugants (1 to 4) with primer combinations specific for the shuttle vector pMVS-V1 replicon (1-kb fragment) and for genomic DNA (gDNA) of *M. thermautotrophicus* ΔH (2.8-kb fragment). N, water negative control; WT, control with wild-type *M. thermautotrophicus* ΔH; M, GeneRuler 1-kb DNA ladder (Thermo Scientific, Waltham, MA, USA). (D) Experimental conditions for the confirmation of conjugation as the mechanism for DNA transfer were (from left to right) DNase I treatment, heat inactivation of E. coli S17-1, conjugation with nonconjugative E. coli NEB stable, and addition of free plasmid DNA directly to *M. thermautotrophicus* ΔH cell culture.

Following our broad approach, we investigated several published protocols (and modified versions) to transfer DNA into *M. thermautotrophicus* ΔH by using our various plasmids and shuttle vectors (Materials and Methods). These DNA transfer protocols included (i) natural competence protocols (16, 24), (ii) chemically/physically induced transformation protocols, such as with elevated calcium and magnesium concentrations in the mineral medium or with low-temperature incubation of precultures to induce stress conditions, (iii) an electroporation protocol (29), and (iv) an interdomain conjugation protocol with E. coli (30). Most attempts did not result in cells with the expected selectable phenotype and, if so, could not be linked to the respective anticipated genotype and appeared to be spontaneously resistant cells. The protocol that finally led to a successful DNA transfer into *M. thermautotrophicus* ΔH was an interdomain conjugation protocol with E. coli S17-1 (Fig. 2 and Materials and Methods), which was a modified version of the protocol for conjugational DNA transfer into Methanococcus maripaludis (30).

To achieve a successful conjugational DNA transfer of the archetype shuttle vector pMVS-V1 from E. coli S17-1 (donor) to *M. thermautotrophicus* ΔH (recipient), we increased the recipient cell concentration to ∼1.6 × 10^9^ cells from a culture in the early stationary growth phase, which is a 5-fold-higher cell concentration than that in the work of Dodsworth et al. (30). Furthermore, we used a spot-mating procedure to allow close physical contact between donor and recipient cells during an incubation period at 37°C on solidified medium plates, which supported metabolic activity of E. coli S17-1 (Fig. 2A), in contrast to direct spread plating as in the work of Dodsworth et al. (30). After resuspending the spot-mated cells (donor + recipient) from the solidified medium plate, we recovered *M. thermautotrophicus* ΔH in liquid mineral medium without any complex medium additions (no organic carbon source) and without antibiotic additions at 60°C under a molecular hydrogen/carbon dioxide atmosphere (Fig. 2B and Materials and Methods). These incubation conditions decreased the viability of E. coli S17-1 to a very minimum, and therefore, no counterselection with antibiotics against donor cells was required. After a short incubation period (3 to 4 h) to recover *M. thermautotrophicus* ΔH under nonselective conditions, the cells were transferred to liquid medium, which contained neomycin for a selective-enrichment step. Importantly, the required incubation period for the cells to grow in this step was key for a successful identification of transconjugants (i.e., recipient cells that received the shuttle vector). Neomycin at a concentration of 250 μg/ml inhibits growth of *M. thermautotrophicus* ΔH for at least 60 h in liquid medium (Text S1B). Therefore, when the cells did not grow within less than 60 h (typically growth appeared after 24 to 48 h in a successful experiment) in the selective-enrichment step, the conjugation experiment was regarded as unsuccessful, because the number of spontaneously resistant cells is considerably higher than potential transconjugants, which renders screening essentially impossible.

### The shuttle-vector DNA confers the observed antibiotic-resistant phenotype and is maintained in *M. thermautotrophicus* ΔH with high segregational stability.

After we found selective growth of putative transconjugant cells, we confirmed the successful DNA transfer into *M. thermautotrophicus* ΔH via two site-specific PCRs, which amplified a fragment of the pMVS-V1 shuttle vector and a fragment of the *M. thermautotrophicus* ΔH genomic DNA, respectively, with liquid cultures derived from individual colonies (Fig. 2C). In addition, we extracted plasmid DNA from several independent *M. thermautotrophicus* ΔH transconjugant cultures, transformed E. coli with this plasmid DNA extract, reextracted the plasmid DNA again from E. coli, and finally, performed restriction-enzyme digestions and Sanger sequencing to confirm the plasmid DNA integrity and sequence, without finding any differences from the original pMVS-V1 shuttle vector (Text S1D and Fig. S4). With different shuttle vectors in independent experiments, we achieved reliable DNA transfer into *M. thermautotrophicus* ΔH with our standard protocol (Materials and Methods), which includes a selective-enrichment step (Fig. 2B). However, this protocol was not suitable to calculate the conjugation frequency, due to the selective-enrichment step. To determine the conjugation frequencies, we performed experiments without the selective-enrichment step but with a prolonged nonselective-recovery step, which resulted in 5 ± 4 colonies (*n *= 6) and from which we calculated conjugation frequencies of approximately 4 × 10^−9^ to 6 × 10^−6^ transconjugants per initial recipients (see equation S1 in Text S1F). With this assessment, we showed that experimental variations considerably influenced the conjugation frequency (Text S1F and Table S1). Nevertheless, when following our standard protocol, a reliable transfer of plasmid DNA was achieved. Once the plasmid DNA was transferred into *M. thermautotrophicus* ΔH, it was maintained with high segregational stability over many cell divisions in an experiment under nonselective growth conditions, and we did not observe loss of pMVS-V1 (Text S1G and Fig. S5).

10.1128/mBio.02766-21.5 FIG S4 (A) Restriction-enzyme digestion of plasmid DNA, which was extracted from three independent E. coli pMVS-V1 enrichment cultures (1 to 3). The E. coli strains were generated with plasmid DNA, which was extracted from genetically modified *M. thermautotrophicus* ΔH. The unique cutter KpnI (K), resulting in one 8.2-kb fragment, and the dual cutter NdeI (N), resulting in 2.7-kb and 5.5-kb fragments, were used. M, GeneRuler 1-kb DNA ladder (Thermo Scientific, Waltham, MA, USA). (B) Exemplified sequence alignment of Sanger sequences from retransformed E. coli plasmid DNA to the original pMVS-V1 sequence (main text and Fig. 1 for further explanations). Red arrows indicate the sequence and its direction. No deletions, insertions, or nucleotide exchanges were detected. Download FIG S4, TIF file, 0.1 MB. Copyright © 2021 Fink et al. 2021 Fink et al. https://creativecommons.org/licenses/by/4.0/ This content is distributed under the terms of the Creative Commons Attribution 4.0 International license.

10.1128/mBio.02766-21.6 FIG S5 (A) PCR analysis of individual colonies from selective solidified medium plates after one selective transfer of pMVS-V1-carrying *M. thermautotrophicus* ΔH in liquid mineral medium. (B) PCR analysis of individual colonies from nonselective solidified medium plates after three nonselective transfers (∼21 to 28 cell divisions) in liquid mineral medium (PCR analysis of the first and second transfer is not shown but gave the same results). A 1-kb fragment results from the specific primer pair for the pME2001 replicon for *M. thermautotrophicus* ΔH (a) and a 1.5-kb fragment from the specific primer pair for *M. thermautotrophicus* ΔH genomic DNA (b). All 16 colonies each result in positive PCR for both primer combinations. Wild-type *M. thermautotrophicus* ΔH (WT) does not result in PCR signal for pMVS-V1, and water as negative control (N) does not result in any PCR signal. M, GeneRuler 1-kb DNA ladder (Thermo Scientific, Waltham, MA, USA). Download FIG S5, TIF file, 0.4 MB. Copyright © 2021 Fink et al. 2021 Fink et al. https://creativecommons.org/licenses/by/4.0/ This content is distributed under the terms of the Creative Commons Attribution 4.0 International license.

10.1128/mBio.02766-21.10 TABLE S1 Example calculations for conjugation frequencies for DNA transfer into *M. thermautotrophicus* ΔH based on equation S1. Download Table S1, DOCX file, 0.01 MB. Copyright © 2021 Fink et al. 2021 Fink et al. https://creativecommons.org/licenses/by/4.0/ This content is distributed under the terms of the Creative Commons Attribution 4.0 International license.

### Free plasmid DNA is not resulting in DNA transfer into *M. thermautotrophicus* ΔH.

By having demonstrated DNA transfer into *M. thermautotrophicus* ΔH, we further analyzed whether this transfer was indeed depending on conjugational DNA transfer from E. coli or whether it was rather by uptake of free DNA under the utilized cultivation conditions during the conjugation protocol (Materials and Methods). E. coli S17-1 donor cells contain a large amount of pMVS-V1, because of the high-copy-number ColE1 replicon, which might be released into the liquid medium from lysing cells. Therefore, we conducted control experiments with free pMVS-V1 plasmid DNA, heat-inactivated E. coli S17-1 cells, and nonconjugative E. coli NEB stable cells that carry pMVS-V1 (Fig. 2D). None of these experiments resulted in DNA transfer into *M. thermautotrophicus* ΔH (Fig. 2D, Text S1H, and Fig. S6). In contrast, DNase I treatment of the E. coli S17-1 donor cells did not negatively influence the success of a conjugational DNA transfer into *M. thermautotrophicus* ΔH (Fig. 2D, Text S1H, and Fig. S6). Thus, we concluded that DNA transfer occurs due to conjugational mobilization activity from E. coli S17-1 to *M. thermautotrophicus* ΔH.

10.1128/mBio.02766-21.7 FIG S6 (A) Enrichment cultures of genetically modified *M. thermautotrophicus* ΔH in 250-μg/ml neomycin-containing selective liquid mineral medium from conjugation/DNA transfer experiments with the standard protocol (1), the DNase I-treated E. coli S17-1 (2), the heat-inactivated E. coli S17-1 (3), and NEB stable as nonconjugative E. coli (4). Slight turbidity in cultures 3 and 4 is caused due to high initial cell count. Enrichment is visible in cultures 1 and 2. (B) Spread-plated *M. thermautotrophicus* ΔH using culture 1 from panel A (standard protocol). Black circles represent colonies, which were used for PCR analysis. (C) Spread-plated *M. thermautotrophicus* ΔH using culture 2 from panel A (DNase I treatment). Black circles represent colonies, which were used for PCR analysis. (D) PCR analysis of the four colonies from panels B and C (1 to 4), wild-type *M. thermautotrophicus* ΔH (5), purified shuttle-vector DNA as positive control (6), and water as negative control (7). A primer combination which amplifies a 1-kb fragment from the pME2001 replicon was used for PCR amplification. M, GeneRuler 1-kb DNA ladder (Thermo Scientific, Waltham, MA, USA). Download FIG S6, TIF file, 0.4 MB. Copyright © 2021 Fink et al. 2021 Fink et al. https://creativecommons.org/licenses/by/4.0/ This content is distributed under the terms of the Creative Commons Attribution 4.0 International license.

### A thermostable β-galactosidase (BgaB) from Geobacillus stearothermophilus is a functional reporter to investigate promoter sequences in *M. thermautotrophicus* ΔH.

**FIG 3:**
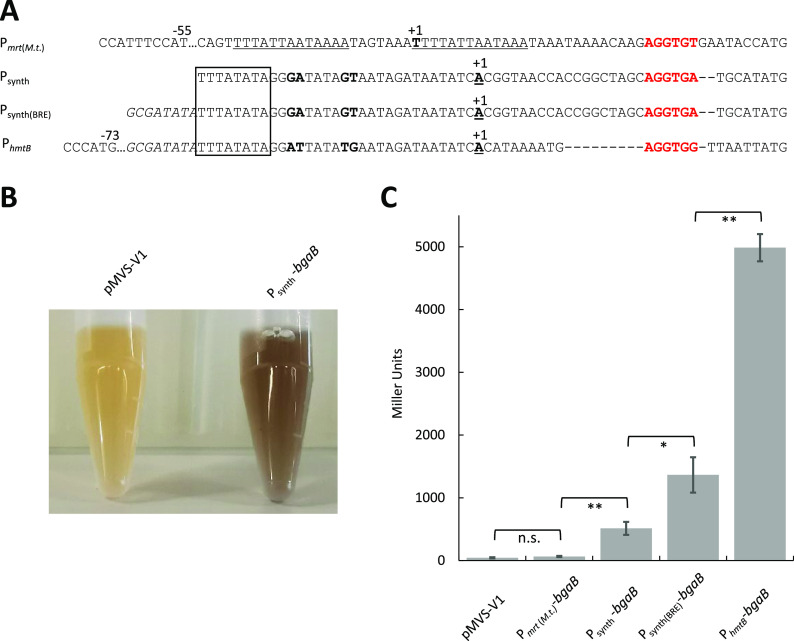
Enzyme activity assays with *M. thermautotrophicus* ΔH strains that carry a thermostable β-galactosidase (BgaB)-encoding gene under the control of four distinct promoter sequences. (A) Sequence alignment of distinct putative promoter sequences that we analyzed for activity to drive the expression of a thermostable β-galactosidase (*bgaB*) gene. Sequence repeats in P*_mrt_*_(_*_M.t._*_)_ are underlined. The transcription start site is indicated by “+1,” highlighted in bold, and underlined. TATA box sequences of P_synth_, P_synth(BRE)_, and P*_hmtB_* are surrounded by a box. BRE sequences are highlighted in italics and ribosome-binding sites in red. Dashes are used as spacers, while dots indicate additional base pairs, which are left out here for visualization. Differences between P_synth_, P_synth(BRE)_, and P*_hmtB_* between the TATA box sequence and transcription start site are highlighted in bold. (B) Qualitative analysis of BgaB activity with S-Gal as chromogenic substance in an *in vitro* assay with cell lysate of empty-vector-carrying *M. thermautotrophicus* ΔH (pMVS-V1) or pMVS1111A:P_synth_-*bgaB*-carrying *M. thermautotrophicus* ΔH (P_synth_-*bgaB*) cells. (C) Quantitative analysis of BgaB activity with ONPG as chromogenic substance in an *in vitro* assay with cell lysate of *M. thermautotrophicus* ΔH strains that carry plasmids with the *bgaB* gene under the control of the four distinct promoters [pMVS-V1, empty-vector control; P*_mrt_*_(_*_M.t._*_)_-*bgaB*, pMVS1111A:P*_mrt_*_(_*_M.t._*_)_-*bgaB*; P_synth_-*bgaB*, pMVS1111A:P_synth_-*bgaB*; P_synth(BRE)_-*bgaB*, pMVS1111A:P_synth(BRE)_-*bgaB*; P*_hmtB_*-*bgaB* pMVS1111A:P*_hmtB_*-*bgaB*]. Average (*n *= 3) with error bars indicating standard deviation. Significance was tested with Student’s *t* test (two-tailed): *, significant difference (*P* < 0.05); **, highly significant difference (*P* < 0.01); n.s., no significant difference (*P* > 0.05).

With a DNA transfer protocol and a functional shuttle vector at hand, we proceeded with adding a genetic cargo (i.e., gene of interest) to the application module of the archetype pMVS-V1 shuttle vector. To enable the analysis of the effects from different promoter sequences on gene expression in *M. thermautotrophicus* ΔH, we decided to implement a reporter gene as our first gene of interest. We chose the *bgaB* gene from *G. stearothermophilus*, which encodes a thermostable β-galactosidase (31). We placed a codon-optimized version of the *bgaB* gene under the control of the nonnative P_synth_ promoter (Materials and Methods). We transferred the resulting shuttle vector pMVS1111A:P_synth_-*bgaB* (Fig. 1B) into *M. thermautotrophicus* ΔH via conjugation. In a qualitative preliminary experiment with cell lysate from pMVS1111A:P_synth_-*bgaB*-carrying *M. thermautotrophicus* ΔH cells (and pMVS-V1-carrying cells as an empty-vector negative control), we found that, indeed, the β-galactosidase BgaB is produced in *M. thermautotrophicus* ΔH and results in a color reaction in an enzyme assay with 3,4-cyclohexenoesculetin-β-d-galactopyranoside (S-Gal) only in the presence of the *bgaB* gene (Fig. 3B and Materials and Methods).

This result sparked us to establish a quantitative β-galactosidase enzyme activity assay with *o*-nitrophenyl-β-d-galactopyranoside (ONPG) as the chromogenic substrate for the β-galactosidase (Text S1I), which allowed us to investigate promoter sequences for their relative *in-vivo* effect on gene expression in *M. thermautotrophicus* ΔH during a growth experiment (Fig. S1I). Overall, we selected four distinct promoter sequences [P_synth_, P_synth(BRE)_, P*_hmtB_*, and P*_mrt_*_(_*_M.t._*_)_] based on our previous results and the peer-reviewed literature and compared the effects of these promoters on gene expression with the established enzyme assay (Fig. 3 and Text S1I) (22, 32). With our optimized quantitative β-galactosidase enzyme activity assay, we found that the P_synth_ promoter, without a transcription factor B recognition element (BRE) sequence, resulted in a significantly higher β-galactosidase enzyme activity (510 ± 50 Miller units) than did the empty-vector control (46 ± 5 Miller units; *P* < 0.01) (Fig. 3C). However, significantly lower enzyme activity was measured with this promoter than with the P_synth(BRE)_ (1,350 ± 140 Miller units; *P* < 0.05) and the P*_hmtB_* (5,000 ± 100 Miller units; *P* < 0.01) (Fig. 3C) promoters, which both contained a BRE sequence (Fig. 3A). The P*_mrt_*_(_*_M.t._*_)_ promoter resulted only in a β-galactosidase activity (65 ± 5 Miller units) that was comparable to and not significantly different from the empty-vector negative control in the enzyme assay (45 ± 5 Miller units) (Fig. 3C). Therefore, this promoter has to be considered inactive under the tested conditions (Fig. 3C). We did not include the commonly used P*_mcrB_*_(_*_M.v._*_)_ promoter for methanogen genetic systems in this comparison, because we already had found that this promoter is not functional in driving the neomycin-selectable marker (Text S1I).

### The metabolism of *M. thermautotrophicus* ΔH can be amended to enable growth on formate as an alternative substrate.

**FIG 4:**
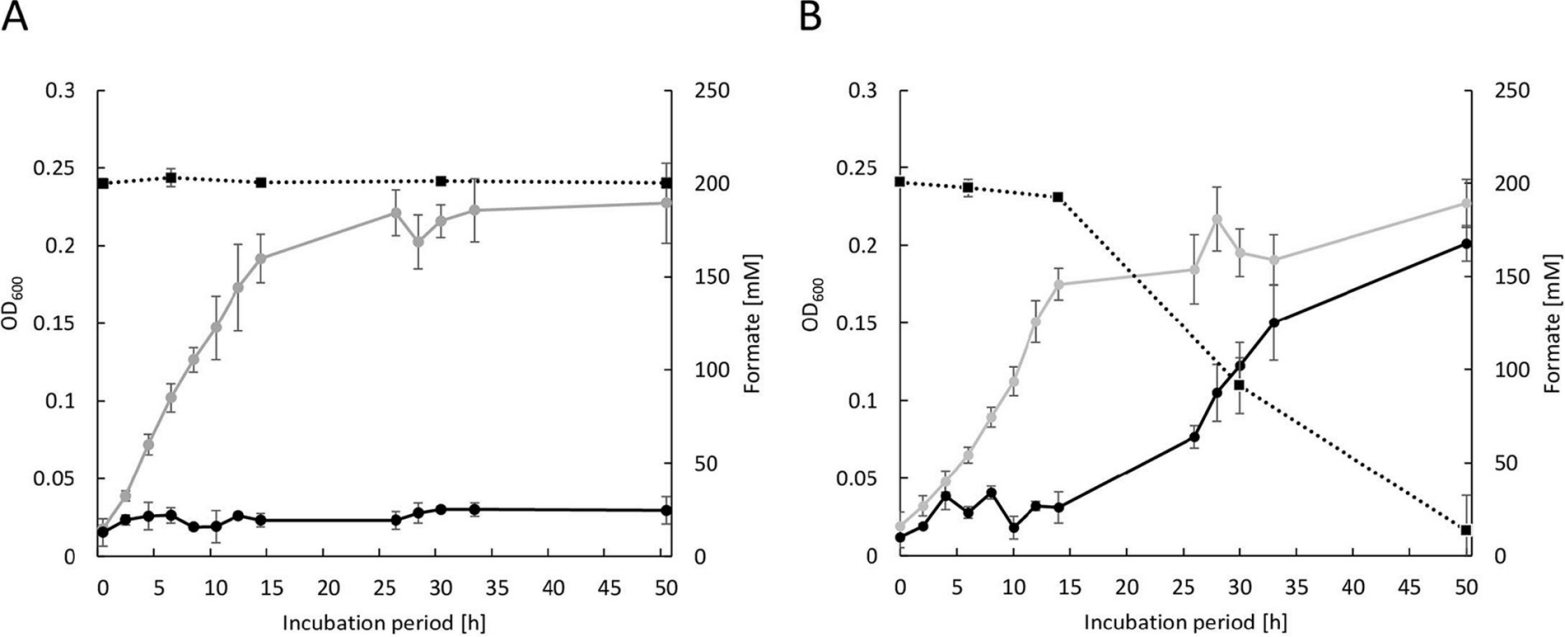
Analysis of genetically modified *M. thermautotrophicus* ΔH strains for growth on formate. (A) Growth behavior of *M. thermautotrophicus* pMVS-V1 on molecular hydrogen and carbon dioxide (gray) and on formate (black) as the carbon and energy source. Average (*n *= 3) with error bars indicating standard deviation. The dotted line indicates the remaining amount of formate in the medium of *M. thermautotrophicus* pMVS-V1 grown on formate determined by HPLC measurements. (B) Growth behavior of *M. thermautotrophicus* pMVS1111A:P*_hmtB_*-*fdh*_Z-245_ with either molecular hydrogen and carbon dioxide (gray) or with formate (black) as the carbon and energy source. Average (*n *= 3) with error bars indicating standard deviation. The dotted line indicates the remaining amount of formate in the medium of *M. thermautotrophicus* pMVS1111A:P*_hmtB_*-*fdh*_Z-245_ grown on formate determined by HPLC measurements.

Formate as the sole carbon and energy substrate can be utilized by several methanogens, such as *Methanococcus* spp. (33), *Methanobacterium* spp., and also *Methanothermobacter* spp. (34). For example, the strain *M. thermautotrophicus* Z-245 can grow with only formate, instead of molecular hydrogen and carbon dioxide, while *M. thermautotrophicus* ΔH is not able to grow with only formate (Fig. 4 and Fig. S8) (34, 35). It was hypothesized by Nölling and Reeve (35) that the genetic reason for this is the missing formate dehydrogenase (*fdh*) operon in the genome of *M. thermautotrophicus* ΔH compared to the same genomic region in *M. thermautotrophicus* Z-245. We argued that we can test this hypothesis *in vivo*, by providing the *fdh* operon as a genetic cargo in the application module of our shuttle vector. Thus, we constructed the shuttle vector pMVS1111A:P*_hmtB_*-*fdh*_Z-245_ that contains the entire *fdh* operon from *M. thermautotrophicus* Z-245, including the genes (in this order) *fdhC*, *fdhA*, and *fdhB* and additionally an open reading frame with unknown function (*orf3*), as indicated in the work of Nölling and Reeve (35), under the control of the constitutive P*_hmtB_* promoter (Fig. 3). In control experiments with *M. thermautotrophicus* Z-245, we confirmed growth of this microbe with either formate or molecular hydrogen and carbon dioxide as substrates (Fig. S8). Growth on formate was possible with *M. thermautotrophicus* ΔH cells that carry pMVS1111A:P_hmtB_-*fdh*_Z-245_ but not with cells that carry the empty-vector control pMVS-V1, and thus, no growth was observed from the small amount of yeast extract that we had added to provide any potentially missing micronutrients (Fig. 4 and Materials and Methods). The formate dehydrogenase-producing strain had a prolonged lag phase with formate but reached a final optical density at 600 nm (OD_600_) comparable to growth on molecular hydrogen and carbon dioxide. Hence, the hypothesis of Nölling and Reeve (35) was proven to be correct.

10.1128/mBio.02766-21.9 FIG S8 Analysis of wild-type *M. thermautotrophicus* Z-245 growth either with molecular hydrogen and carbon dioxide (black solid line) or with formate (gray solid line) as the carbon and energy source. Average (*n *= 3) with error bars indicating standard deviation. Download FIG S8, EPS file, 1.2 MB. Copyright © 2021 Fink et al. 2021 Fink et al. https://creativecommons.org/licenses/by/4.0/ This content is distributed under the terms of the Creative Commons Attribution 4.0 International license.

## DISCUSSION

Here, we reported a robust method for genetic manipulation of *M. thermautotrophicus* ΔH (Fig. 2), including replicating shuttle vectors, which we based on the cryptic plasmid pME2001 (12). To achieve this, in a first step, we investigated different plating protocols to obtain clonal populations. Importantly, while we achieved plating efficiencies of up to 100%, the plating conditions considerably influenced the outcome over a range of 3 orders of magnitude (see Text S1J in the supplemental material). Thus, for specific purposes the plating technique has to be carefully considered to avoid misleading results, such as false interpretations of DNA transfer events. At a too-high plating efficiency, spontaneously resistant cells might overgrow genetically modified *M. thermautotrophicus* ΔH.

In general, it was noticeable that spontaneously resistant *M. thermautotrophicus* ΔH cells appeared readily with the antibiotics simvastatin and neomycin. Once those spontaneously resistant cells appeared, they were not inhibited or delayed in growth in subsequent transfers in liquid medium as well as on solidified medium plates. This has been already reported with neomycin for *M. thermautotrophicus* ΔH (19), *M. maripaludis*, and Methanococcus vannielii (36). However, in comparison to *M. maripaludis* for which spontaneously neomycin-resistant colonies appeared to be smaller than genetically modified colonies (which were found to carry a neomycin-selectable marker) (37), we cannot see this difference in colony size for *M. thermautotrophicus* ΔH. Therefore, we implemented a selective-enrichment step in liquid medium, which provided enough time for growth of the genetically modified *M. thermautotrophicus* ΔH, while at the same time it excluded the onset of growth of the spontaneously resistant cells by substrate limitation in the gas phase, because those cells appeared only after a longer incubation period (Fig. S2). After the selective-enrichment step, the genetically modified *M. thermautotrophicus* ΔH cells outnumbered the spontaneously resistant cells sufficiently to obtain and select genetically modified individual colonies on selective solidified medium plates.

Yet, we were not successful with genetically manipulating *M. thermautotrophicus* ΔH when we fused various potential selectable markers with the P*_mcrB_*_(_*_M.v._*_)_ promoter, which is commonly utilized in other methanogen genetic systems. However, after we switched to the P_synth_ promoter to drive the neomycin-selectable marker, positive selection of shuttle vector-carrying cells with neomycin was possible in combination with the selective-enrichment step. Taken together, we believe that the most important parameters for the successful implementation of our genetic tools for *M. thermautotrophicus* ΔH, compared to previous attempts during the last four decades, were (i) the construction of the shuttle vector with restriction/ligation-independent cloning to fuse the pME2001 replicon with the other modules precisely at the IF5 sequence, to not interrupt any open reading frame or potential sequence of the origin of replication, (ii) the adaptations to the published conjugation protocol for *Methanococcus* spp. by Dodsworth et al. (30), specifically the applied temperature, medium, and headspace gas conditions during the spot-mating procedure, in combination with the selective-enrichment step with limited gas supply that facilitated the selection for genetically modified cells over spontaneously resistant cells, and (iii) the utilization of a constitutive native promoter sequence, which was demonstrated to be functional *in vitro* and in other thermophilic archaea, because the classical P*_mcrB_*_(_*_M.v._*_)_ promoter turned out to be inactive in *M. thermautotrophicus* ΔH.

With the reporter gene *bgaB*, which encodes a thermostable β-galactosidase, we further confirmed the activity of this P_synth_ promoter, as well as the activity of the P_synth(BRE)_ and P*_hmtB_* promoters, which are all based on the promoter region upstream of the *hmtB* gene of *M. thermautotrophicus* ΔH. The BRE sequence upstream of the P_synth_ sequence, which we had implemented in P_synth(BRE)_, and differences in the transcription-initiation region in P*_hmtB_* compared to both P_synth_ and P_synth(BRE)_ influenced the promoter strength significantly (Fig. 3). This was not surprising, because the critical role of the BRE sequence in archaea is well known, and similar observations were made with archaeal promoters in Saccharolobus solfataricus and Haloferax volcanii, where promoter strength was influenced by the presence or absence of a BRE sequence (38, 39). Furthermore, modifications in the transcription-initiation region in the promoter sequence were shown to influence the strength of gene expression in Sulfolobus acidocaldarius and S. solfataricus (38, 40). Thus, we now have established constitutive promoters of different strength. The thermostable β-galactosidase as a reporter provides an adequate basis to further investigate promoter sequences of *M. thermautotrophicus* ΔH and to establish inducible-promoter systems.

In addition to demonstrating heterologous production of functional β-galactosidase enzymes in *M. thermautotrophicus* ΔH, we demonstrated the production of an active formate dehydrogenase enzyme complex from the *fdh* operon from *M. thermautotrophicus* Z-245 (35), which amended the metabolism of *M. thermautotrophicus* ΔH for the ability to utilize formate as an alternative growth substrate (Fig. 4). These results are a cornerstone for heterologous and homologous (over)expression of genes in *M. thermautotrophicus* ΔH and enable researchers to further expand the genetic toolbox with methodology for chromosomal gene deletions and integrations such as allelic exchange, markerless mutagenesis (41), and CRISPR/Cas technology (42). We will now be able to target modifications in the metabolism of *M. thermautotrophicus* ΔH not only on the substrate but also on the product side. The possibility to change the product spectrum of hydrogenotrophic methanogens has been demonstrated already for *M. maripaludis* with a genetic modification that resulted in geraniol production (43). Our genetic tools for heterologous gene expression enable us to broaden the product spectrum of *M. thermautotrophicus* ΔH and to utilize this industrially relevant and robust microbe for power-to-chemicals (i.e., power-to-x) applications.

## MATERIALS AND METHODS

### Microbial strains, media, and cultivation conditions.

*M. thermautotrophicus* ΔH (DSM 1053), *M. thermautotrophicus* Z-245 (DSM 3720), and *M. marburgensis* (DSM 2133) were obtained from the DSMZ (Braunschweig, Germany) and were cultivated in mineral medium according to basic principles for methanogen cultivation as stated in the work of Balch et al. (44), with modifications to the medium composition when required and with adjustments to state-of-the-art anaerobic handling equipment. The mineral medium contained (per liter) sodium chloride, 0.45 g; sodium hydrogen carbonate, 6.00 g; dipotassium hydrogen phosphate, 0.17 g; potassium dihydrogen phosphate, 0.23; ammonium chloride, 0.19 g; magnesium chloride hexahydrate, 0.08 g; calcium chloride dihydrate, 0.06 g; ammonium nickel sulfate, 1 ml (0.2 wt%); iron(II)chloride pentahydrate, 1 ml (0.2 wt%); resazurin indicator solution, 4 ml (0.025 wt%); and trace element solution, 1 ml (10-fold as stated in the work of Balch et al. [44]). All chemicals were *per analysis* grade. No vitamins were added. For the formate growth experiments, the mineral medium was supplemented with 200 mM sodium formate, 10 μM sodium molybdate, 1 μM sodium selenite, and 0.125 wt% yeast extract for all tested strains, including the empty-vector-carrying *M. thermautotrophicus* ΔH strain, to provide a source of potentially limiting micronutrients. The medium was gassed using N_2_/CO_2_ (80/20 vol%) to eliminate dissolved oxygen. The pH value was adjusted to 7.2 using hydrochloric acid. As reducing agent and sulfur source, 0.5 g/liter cysteine hydrochloride, and for solid mineral medium additionally 0.3 g/liter sodium sulfide monohydrate, was added. Afterward, the mineral medium was dispensed into serum bottles inside an anaerobic chamber with a 100% N_2_ atmosphere (UniLab Pro Eco; MBraun, Garching, Germany). The headspace of the serum bottles was exchanged to 200 kPa H_2_/CO_2_ (80/20 vol%) and autoclaved (100 kPa, 20 min, 121°C). For the formate growth experiments, the headspace of the serum bottles was exchanged to 152 kPa H_2_/CO_2_ (80/20 vol%) as positive control, which provides the same electron equivalents as 200 mM formate, or 152 kPa N_2_/CO_2_ (80/20 vol%), when formate was the substrate. All *Methanothermobacter* strains in liquid medium were incubated at 60°C with shaking at 150 rpm (Lab Companion ISS-7100R; Jeio Tech, Republic of Korea). For cultivation of genetically modified *M. thermautotrophicus* ΔH strains, 250 μg/ml neomycin sodium salt was added. For solidified mineral medium, 1.5 wt% Bacto agar (BD Life Science, Berkshire, United Kingdom) was added as a supplement prior to autoclaving. Afterward, solidified medium plates were poured and dried for 2 h inside the anaerobic chamber. *M. thermautotrophicus* ΔH was applied to solidified medium plates by spot plating, spread plating, or pour plating. For spot plating, 50 μl of *M. thermautotrophicus* ΔH culture was spotted on a solidified medium plate. Incubation was started after the drop was completely absorbed. For spread plating, 50 μl of diluted or undiluted liquid culture was applied to a solidified medium plate and spread out with a Drigalski spatula until the liquid was completely absorbed. For pour plating, 5 ml mineral medium containing 0.8 wt% Bacto agar (soft agar) was mixed with a liquid *M. thermautotrophicus* ΔH culture and poured on top of a solidified medium plate, which contained 1.5 wt% Bacto agar. For better gas-solid mass transfer and to avoid sealing of the plates by water, paper clips were added to the petri dish prior to incubation in a custom-made stainless-steel jar (Raff + Grund, Freiburg am Neckar, Germany) inspired by the work of Balch et al. (44). The gas phase of the stainless-steel jar was exchanged to 200 kPa of H_2_/CO_2_/H_2_S (79.9/20/0.1 vol%). The pressurized anaerobic jar was incubated without shaking at 60°C (Memmert, Schwabach, Germany).

For general cloning and gene manipulation, E. coli NEB stable (New England Biolabs, Frankfurt/Main, Germany) was used. E. coli S17-1 for conjugational DNA transfer was kindly provided by Wolfgang Wohlleben of the Department for Biotechnology at the University of Tübingen, Germany (45). E. coli BL21(DE3) with pME2508 (Archaea Center of the University of Regensburg, Germany) was used to produce recombinant pseudomurein endoisopeptidase (PeiP) enzyme. E. coli was cultivated in LB medium, which contained (per liter) sodium chloride, 10 g; tryptone, 10 g; and yeast extract, 5 g, and which was supplemented with appropriate amounts of chloramphenicol (30 μg/ml), ampicillin (100 μg/ml), or kanamycin (50 μg/ml). For cultivation of E. coli S17-1, trimethoprim (10 μg/ml) was added to stabilize the genome-integrated *tra* module, which is responsible for mobilization of plasmid DNA (45). Solidified LB medium plates contained 1.5 wt% of Kobe I agar (Carl Roth, Karlsruhe, Germany) and were incubated at 37°C. Liquid E. coli cultures were incubated at 37°C with shaking at 150 rpm.

### Molecular cloning and plasmid construction.

All primers, gBlock DNA fragments (IDT, Coralville, IA, USA), and plasmids used in this study are summarized in Tables 1 to 3. PCR was performed with Q5 Hot Start high-fidelity polymerase (NEB, Ipswich, MA, USA) according to the manufacturer’s guidelines and with the required primer combinations (Table 1). Primer concentrations were reduced 10-fold, and elongation time was prolonged by 1 min. Resulting PCR products were DpnI digested when required and purified using a PCR purification kit (Qiagen, Hilden, Germany). For initial fusion of the first shuttle-vector construct pSV1_1, as described below, we used the Gibson Assembly Ultra kit (Synthetic Genomics, La Jolla, CA, USA). All follow-up constructs were assembled with Gibson Assembly master mix (New England Biolabs [NEB], Ipswich, MA, USA) or restriction/ligation cloning with the aid of the implemented modular restriction enzyme-recognition sites (Fig. 1). E. coli cells were transformed with DNA via chemical transformation by following a standard heat shock protocol (46). All plasmids were confirmed by Sanger sequencing (MPI Genomics Center, Tübingen, Germany).

**TABLE 1:** List of primers used in this study

Name	Purpose^*a*^	Sequence (5′→3′)	Reference
Gib_CF1	pCF203	ATAAAATGCTTGGGAGATGACGCCCGCCCCAC	This study
Gib_CF2	pCF203	ATAATCTCCTCTATTTCCATGAGAATCACTCCTATTTTTTTGATATATACATCATAACATTAC	This study
Gib_CF3	pCF203	AAAAAATAGGAGTGATTCTCATGGAAATAGAGGAGATTATAGAGAAAGTTGCTAGG	This study
Gib_CF4	pCF203	TGCGGGTCGTGGGGCGGGCGTCATCTCCCAAGCATTTTATGAGCCCTAGC	This study
Gib_CF5	pSB1	GCCGGTGGTTACCGTGATATTATCTATTACTATATCCCTATATAAAGAATACTCAAAAAATGGGC	This study
Gib_CF6	pSB1	GTAATAGATAATATCACGGTAACCACCGGCTAGCAGGTGATGCATATGGCTAAAATGAGAATATCAC	This study
Gib_CF7	pSV1_1	TATTTTGAATCCATTGCGTTGCGCTCACTG	This study
Gib_CF8	pSV1_1	TGGGCGGCCGGCCGCGTTAATATTTTGTTAAAATTCGCGTTAAATTTTTGTTAAATCAG	This study
Gib_CF9	pSV1_1	AAATATTAACGCGGCCGGCCGCCC	This study
Gib_CF10	pSV1_1	TGCATTTTTTTGCGGCGCGCCCTGACA	This study
Gib_CF11	pSV1_1	AGCGCAACGCAATGGATTCAAAATAGATTCATAATGGAGTCATCCACG	This study
Gib_CF12	pSV1_1	TCAGGGCGCGCCGCAAAAAAATGCAAATAAAATTTGGGGTGG	This study
Res_CF1	pSV1_2	CGTACTGCAGCGATCGCGGTCATATGGATACAGCGGCC	This study
Res_CF2	pSV1_2	GTTATGGATTATAAGCGGCCGGC	This study
Gib_CF13	pMVS1111A:P_synth_-*bgaB*	CCACCCTGCCACCCCAAATTTTATTTGCATTTTTTTGCGGGTTAATTAAGCCTGGAGGAATGCCTTTATATAGG	This study
Gib_CF14	pMVS1111A:P_synth_-*bgaB*	TTTATATATTTTTAATTCACTGGGGGCAATTCTGTCAGGGCGCGCCTGGGGTCGTGCGCTC	This study
Gib_CF15	pMVS1111A:P*_hmtB_*/P*_mrt_*_(_*_M.t_*_.)_-*bgaB*	CGGCTCTAGCTATGTCCGATC	This study
Gib_CF16	pMVS1111A:P*_hmtB_*/P*_mrt_*_(_*_M.t_*_.)_-*bgaB*	CACTGGGGGCAATTCTGTCAG	This study
Gib_CF21	pCF201	AATACAAGAAAGGCGCGCCAAATCATTATATAGGACCTTGATAAAATTTTTTAGAGGGC	This study
Gib_CF22	pCF201	ACCTGACGTGTGGCCGGCCCGATTCAAATATAACAGCCGTTATAACACCGC	This study
Gib_CF23	pCF201	AATCGGGCCGGCCACACGTCAGGTGGCACTTTTCG	This study
Gib_CF24	pCF201	CATCCACGGATGCGATCGCCTAAGAAACCATTATTATCATGACATTAACCTATAAAAATAGGC	This study
Gib_CF17	pCF202	AAAAAATAGGAGTGATTCTCATGGCTAAAATGAGAATATCACCGGAAT	This study
Gib_CF18	pCF202	TGCGGGTCGTGGGGCGGGCGCTAAAACAATTCATCCAGTAAAATATAATATTTTATTTTCTCCCAAT	This study
Gib_CF19	pCF202	GATATTCTCATTTTAGCCATGAGAATCACTCCTATTTTTTTGATATATACATCATAACATTAC	This study
Gib_CF20	pCF202	TACTGGATGAATTGTTTTAGCGCCCGCCCCACG	This study
Res_LM1	pMVS1111A:P*_hmtB_*-*fdh*_Z-245_	ATCGGCTTAGGCGCGCCTGCTCATCGTCAATTCTAGTAGAGTCATGAATCATTATGCAGG	This study
Res_LM2	pMVS1111A:P*_hmtB_*-*fdh*_Z-245_	GCTTAGCGCATTAATTAACCGCCCATTTTTTGAGTATTC	This study
Gib_LM1	pLM201	TAACAGCGGCGCTATCAAGGTCCTGCATAATGATTCATGACGCCCGCCCCACG	This study
Gib_LM2	pLM201	TTTGCCGTATCTGCAGGCGATTTAAAAGATGATCCCATGAGAATCACTCCTATTTTTTTG	This study
Gib_LM3	pLM201	TTATGATGTATATATCAAAAAAATAGGAGTGATTCTCATGGGATCATCTTTTAAATCGCC	This study
Gib_LM4	pLM201	TCCTTTCGGTCGGGCGCTGCGGGTCGTGGGGCGGGCGTCATGAATCATTATGCAGGACC	This study
Gib_LM5	pLM202	ATCCTATATAAATATATCGCTAATTTTAAGGTTTTTCTGAGCCATCGGTTGGTTCATGGGTTAATTAAGAATACTCAAAAAATGGGCG	This study
Gib_LM6	pLM202	CGATATATTTATATAGGATTATATGAATAGATAATATCACATAAAATGAGGTGGTTAATTATGGGATCATCTTTTAAATCGCCTGCAG	This study
Seq_CF1	Specific for gDNA *M. t.*1.5 kb	CCACCAGTTCGACTCCCTGG	This study
Seq_CF2	Specific for gDNA *M. t.*1.5 kb	CTGTTAAAGGCGGGGGTGG	This study
Seq_CF3	Specific for gDNA *M. t.* 2.8 kb	CTTGGGTGATGATGGGATGTATTG	This study
Seq_CF4	Specific for gDNA *M. t.* 2.8 kb	CGAGGAGAAACACATCCAGCTG	This study
Seq_CF5	Specific for pME2001 replicon	GTTAATCCAGCACATCCTCC	This study
Seq_CF6	Specific for pME2001 replicon	CCTGTCCAACTTATACCTTTGG	This study
Seq_CF7	Analysis of *bgaB* constructs	CCCCATAACATCGGCACAGTAC	This study
Seq_CF8	Analysis of *bgaB* constructs	CCTGGCTGGGGTTAATAAATGTTG	This study
Seq_LM1	Analysis of *fdh*_Z-245_ constructs	GATTTCTGGAATCCGCCATGGG	This study
Seq_LM2	Analysis of *fdh*_Z-245_ constructs	CTAATAGTCGCCGATCCAAG	This study
Seq_LM3	Analysis of *fdh*_Z-245_ constructs	GGTTCCTGGCTTGAATG	This study
Seq_LM4	Analysis of *fdh*_Z-245_ constructs	GAGAAGCAAAGGATGACTG	This study
Seq_LM5	Analysis of *fdh*_Z-245_ constructs	CAGCACCCATCTTATTCG	This study
Seq_LM6	Analysis of *fdh*_Z-245_ constructs	GCAGTTAAGAAGGGTTCG	This study
Seq_LM7	Analysis of *fdh*_Z-245_ constructs	GGCTCCGTTATAAGGGTTG	This study
Seq_LM8	Analysis of *fdh*_Z-245_ constructs	CTGAATGGATCGAGAAAGG	This study
Seq_LM9	Analysis of *fdh*_Z-245_ constructs	CATTCTTTCGAGATGGAAG	This study
Seq_LM10	Analysis of *fdh*_Z-245_ constructs	CCTATATTCGCATTCGTGG	This study
Seq_LM11	Analysis of *fdh*_Z-245_ constructs	ATGTTTGCCACACTGTG	This study
Seq_LM12	Analysis of *fdh*_Z-245_ constructs	GGTGGGGTTTTGGTGTGCG	This study

a Abbreviations: gDNA, genomic DNA; *M. t.*, *M. thermautotrophicus*.

**TABLE 2:** List of gBlocks used in this study

Name	Sequence (5′→3′)	Reference
gBlock P*_mcrB_*-*pac*-T*_mcr_*	GGTACCGAAAAGTGCCACCTGACCGATGGCCGGCCGCCCATTTTTTGAGTATTCAAATTCAAATTATTGTGTTATTAACATCTTATATATAAACTTTTCTATTTAATGTTAATGAAAAAGTGAATATATATACATAGAGTAATGTTATGATGTATATATCAAAAAAATAGGAGTGATTCTCATGACCGAGTACAAGCCCACCGTTAGGCTCGCAACCAGGGATGATGTTCCCAGGGCAGTTAGGACCCTCGCAGCAGCATTCGCAGATTACCCCGCAACCAGGCACACCGTTGATCCCGATAGGCACATAGAGAGGGTTACCGAGCTCCAGGAGCTCTTCCTCACCAGGGTTGGTCTCGATATAGGTAAGGTTTGGGTTGCAGATGATGGTGCAGCAGTTGCAGTTTGGACCACCCCCGAGTCAGTTGAGGCAGGTGCAGTTTTCGCAGAGATAGGTCCCAGGATGGCAGAGCTCTCAGGTTCAAGGCTCGCAGCACAGCAGCAGATGGAGGGTCTCCTCGCACCCCACAGGCCCAAGGAGCCCGCATGGTTCCTCGCAACCGTTGGTGTTTCACCCGATCACCAGGGTAAGGGTCTCGGTTCAGCAGTTGTTCTCCCCGGTGTTGAGGCAGCAGAGAGGGCAGGTGTTCCCGCATTCCTCGAGACCTCAGCACCCAGGAACCTCCCCTTCTACGAGAGGCTCGGTTTCACCGTTACCGCAGATGTTGAGTGCCCCAAGGATAGGGCAACCTGGTGCATGACCAGGAAGCCCGGTGCATGACGCCCGCCCCACGACCCGCAGCGCCCGACCGAAAGGAGCGCACGACCCCATGGCTCCGACCGAAGCCACCCGGGGCGGCCCCGCCGACCCCGCACCCGCCCCCGAGGCCCACCGCGGGGGACACACCGAACACGCCGACCCTGCTGAACACGCGGCGCAGTTCGGTGCCCAGGAGCGGATCGGGAATTAATTCGAAGCTGCTGGTGAAAGAGACCCTATCTTACCTGCTAAAATCTAAGTTAATTACTAATTTATTATTAATTTATTATTAGATTGGGCAAAATAGTAAAAGAAAACTAAAGGAAACCTAATATGGTTTCCTTTTTTTATATATTTTTAATTCACTGGGGGCAATTCTGTCAGGGCGCGCCTTCGGGCCATCGGGCCC	This study
gBlock P*_hmtB_*-PacI-*bgaB*	CGGCTCTAGCTATGTCCGATCAATCTTAATTAAGCCTGGAGGAATGCCCCCATGAACCAACCGATGGCTCAGAAAAACCTTAAAATTAGCGATATATTTATATAGGATTATATGAATAGATAATATCACATAAAATGAGGTGGTTAATTATGAACGTTCTCAGTTCCATCTGCTATGGGGGGGATTACAAC	This study
gBlock P*_mrt_*_(_*_M.t_*_.)_-PacI-*bgaB*-cor	CGGCTCTAGCTATGTCCGATCAATCTTAATTAAGCCTGGAGGAATGCCCCATTTCCATGGATTATCGCTGGCAATCCCATAACCCCATCAGTTTTATTAATAAAATAGTAAATTTTATTAATAAATAAATAAAACAAGAGGTGTGAATACCATGAACGTTCTCAGTTCCATCTGCTATGGGGGGGATTACAAC	This study
gBlock codon-optimized *bgaB*	CTGACAGAATTGCCCCCAGTGAATTAAAAATATATAAAAAAAGGAAACCATATTAGGTTTCCTTTAGTTTTCTTTTACTATTTTGCCCAATCTAATAATAAATTAATAATAAATTAGTAATTAACTTAGATTTTAGCAGGTAAGTGGGGTCGTGCGCTCCTTTCGGTCGGGCGCTGCGGGTCGTGGGGCGGGCGCTAGACCTTGCCGGCTTCGTCGTGTTCCCTAAGGACAGCGACGTCGACGCCCTGAATCCTGAGTTCACCCCCCCTGAAGCATTTGCCATCTATCATATTCTGGTAGATCTTATCTTCCGGAAGGGAGAGTGTGACCTCATAGTCGTTGTGGTTAATTATAATAAGGTACTTCCATTCATCGGTCTCCCTCTGCTGAACTTCGACATTCTCAGCAACCTCCAGTATAGGATTTATGTGGTGTTTAGCAAACACCTGTTCGAGAAGCCTGCCAAGGTAGTTGCTGTCAGGGTATGTTCCTACGTATATGCCCTCCCCCTTTCCGTAGCAGTTCCTGGTAACAGCAGGAAGGCCGGCATACCAATCACCTTTGAATGTGGCGAGAGGCTCAGCACCTTCCAGCCTTATTATATCGGCCCATGTGGTACAGTCATACTCGCCGTCGTTTGAGTAGATCTTATTCACCTTTGTCTCGGGATAAGGAACGAATTCCTCCACGAAGATGCCGAGAATGTCCCTCAGCGGTCCTGGATATCCCCCGAGGTGCACTCTATCGTTCTCATCGACTATCACACTGAAAAAGCTTACAATCAGGGTTCCGCCGTTTGCGACAAACTGCCTAAGGTTCTCATCTTCTCCCTCTTTCACCATATACAGCATCGGTGCAATAACAACCTTATATTTTGTGAGATCGTCGGACGGTCTTACAAAGTCGACTGCTATGTTTCTCTTGTAAAGCTCTCTATAATATGCCTCTACTATGGGAATATATCTGAGCTTGTTGTGCGGTTTGGAACTGAGCTCAACTGCCCACCAGTTTTCCCAGTCAAAGATAATTGCCACCTCTGCCTTTATTCTACTCCCCACGAGGCAGTCAAGTTTTTTCAGCTCCTGGCCAAGCTGGGTAACTTCCCTGTATATTCTATTGTTTTCGTTAAGAAAGTGGGGCACCATTGCTCCGTGAAACTTCTCAGCTCCTGCTCTGGACTGCCTCCACTGAAAGAACATTATCCCATCGGCACCCCTGGCGATTGTTGCGTAACTCCAGAGTCTCATAACCCCCGGCGGCTTTGGCACATTGATATCTCTCCAATTAACGTGACTGGTGACCTGCTCCATAAGAATGAACGGCTGCCCCTTCCTAAGTGACCTCATGAGGTCATTCATCATTGCGTGCTGTATAGGGAGTCCCTCCCTGGGATCTGGGTAGCTATCCCAGGTAACGATATCTACGTGCTGAGCCCACTGAAAGTAGTTGAGTGGCTTGAATGATCCCATGAAATTTGTGGAGACCGGGATATCGGGGGTTACTTCCCTGAGGATCTCCTTTTCTGTAAGGAAGAGTTTGAGGATTGAATCATTCATGAATCTGTAGTAATCAAGCTCCTGGCTGGGGTTAATAAATGTTGGTGCCTTCCTAGGGGGATTAATCTCATCCCAGTGGTTATATCTCTGGCCCCAGAAGTTTGTACCCCATCTTTCATTAAGTTCATCAATGGTCTTATACCTTTCTTTAAGCCATTTTCTGAAAGCAACTGCGCAATTCTCACAGAAACACTTACTTACATGGCAAGCGTATTCGTTATTTACGTGCCACATTTTGAGGGCTGGATGATTTTTGTATCTCTCAGCTATAGCCCTTACCAGCCTCTTTATATGTGTTATAAGCTGAGGGTGATTTGGGCAATAATGCTGTCTACTCCCGAAACTCAGTATCACACCGGACTCGTCAATAGGGAGTGAATCAGGGTATTTCTTCACGAACCAGGCGGGTGTGGTTGCGGTGGCGGTCCCCAGATTTATGTATACCCCATGATCGTAGAGGATGTCTATCACTTTGTCGAGCCATTCAAAATCAAATACACCGTCTGATGGCTCGATTTTGGACCAGCTAAAGATTCCGAGTGAAACAAGATTAACACCGGCCTTCTGCATAAGTTTTGCGTCCTCGTACCATATCTCCTCGGGCCACTGTTCTGGGTTGTAATCCCCCCCATAGCAGATGGAACTGAGAACGTTCATATGCATCACCTGCTAGCCGGTGGTTACCGTGATATTATCTATTACTATATCCCTATATAAAGGCATTCCTCCAGGCTTAATTAAC	This study

**TABLE 3:** List of plasmids used in this study

Name	Function	Reference	*M. thermautotrophicus* strain^*a*^
pMTL83151	Shuttle vector for *Clostridia* spp.	Heap et al. (27)	-
pMU131	Shuttle vector for *Thermoanaerobacter* spp.	Shaw et al. (23)	-
pME2001	Cryptic plasmid of *M. marburgensis*	Bokranz et al. (50)	-
pBBR1-MCS2	Standard cloning vector in E. coli	Kovach et al. (51)	-
pUC19	Standard cloning vector in E. coli	Yanisch-Perron et al. (52)	-
pYS3	Shuttle vector for Pyrococcus furiosus including Sim^r^	Waege et al. (21)	-
pME2508	PeiP production in E. coli	Luo et al. (48)	-
pCF200	pUC57 vector including synthesized P*_mcrB_*_(_*_M.v._*_)__Pur^r^ codon-optimized for *M. thermautotrophicus*, T*_mcr_*	This study	-
pCF201	pUC19 vector including native *M. thermautotrophicus* Z-245 *fdh*_Z-245_ operon with putative promoter region	This study	-
pLM201	Exchange of Neo^r^ to coding region of *fdh*_Z-245_ from pCF201 in pCF204	This study	-
pLM202	Exchange of P*_mcrB_*_(_*_M.v._*_)_ to P*_hmtB_* in pLM201	This study	-
pCF203	Exchange of Pur^r^ to Sim^r^ in pCF200	This study	-
pCF204	Exchange of Pur^r^ to Neo^r^ in pCF200	This study	-
pCF404	pUC57 including 1 kb up- and downstream of annotated *pyrF* gene (MTH_RS00570) and P*_mcrB_*_(_*_M.v._*_)__Pur^r^	This study	-
pCF407	Exchange of P*_mcrB_*_(_*_M.v._*_)__Pur^r^ to P*_mcrB_*_(_*_M.v._*_)__Neo^r^ in pCF404	This study	-
pSB1	Exchange of P*_mcrB_*_(_*_M.v._*_)_ promoter to P_synth_ in pCF407	This study	-
pSV1_1	Shuttle vector construct containing P*_mcrB_*_(_*_M.v._*_)__Sim^r^ and pBBR1MCS2 backbone and pME2001 replicon	This study	-
pSV1_2	Shuttle vector construct containing P*_mcrB_*_(_*_M.v._*_)__Sim^r^ and pMTL backbone and pME2001 replicon	This study	-
pSV1_3	Shuttle vector construct containing P*_mcrB_*_(_*_M.v._*_)__Neo^r^ and pMTL80151 backbone and pME2001 replicon	This study	-
pMVS-V1	Shuttle vector construct containing P_synth__Neo^r^ and pMTL backbone and pME2001 replicon	This study	x
pMVS1111A:P_synth_-*bgaB*	Shuttle vector construct pMVS-V1 including β-galactosidase (*bgaB*) gene and promoter P_synth_	This study	x
pMVS1111A:P*_hmtB_*-*bgaB*	Shuttle vector construct pMVS-V1 including β-galactosidase (*bgaB*) gene and promoter P*_hmtB_*	This study	x
pMVS1111A:P*_mrt_*_(_*_M.t._*_)_-*bgaB*	Shuttle vector construct pMVS-V1 including β-galactosidase (*bgaB*) gene and promoter P*_mrt_*_(_*_M.t._*_)_	This study	x
pMVS1111A:P_synth(BRE)_-*bgaB*	Shuttle vector construct pMVS-V1 including β-galactosidase (*bgaB*) gene and promoter P_synth(BRE)_	This study	x
pMVS1111A:P*_hmtB_*-*fdh*_Z-245_	Shuttle vector construct pMVS-V1 including *fdh*_Z-245_ operon from *M. thermautotrophicus* Z-245 and promoter P*_hmtB_*	This study	x

a -, strain not available; x, strain available.

pCF200, which contains the puromycin acetyltransferase (*pac*) gene (Pur^r^) from Streptomyces alboniger as a codon-optimized version for *M. thermautotrophicus* ΔH under the control of the P*_mcrB_*_(_*_M.v._*_)_ promoter and the T*_mcr_* terminator from Methanococcus voltae (47), was completely synthesized (BioCat, Heidelberg, Germany). The *pac* gene in pCF200 was exchanged to the 3-hydroxy-3-methylglutaryl-coenzyme A reductase (HmgA)-encoding gene (Sim^r^) from Thermococcus kodakarensis by using pYS3 (21) and pCF200 as the templates for Gibson Assembly, resulting in pCF203. pCF203, pME2001 (extracted from wild-type *M. marburgensis*), and pBBR1-MCS2 (Addgene catalog no. 85168) were used as the templates for Gibson Assembly with the Gibson Assembly Ultra kit (Synthetic Genomics, La Jolla, CA, USA) and resulted in the putative shuttle vector pSV1_1. pSV1_1 was the basis for further shuttle vectors. For the introduction of a high-copy-number replicon for E. coli, a *tra* region for plasmid mobilization, and an additional AsiSI restriction enzyme-recognition sequence, the pBBR1-MCS2 backbone was exchanged to the E. coli vector backbone from pMTL83151 (27), including Cam^r^, ColE1, and the *tra* minigene for mobilization, via Gibson Assembly resulting in pSV1_2. To implement the thermostable neomycin phosphotransferase gene (Neo^r^) (28), the Pur^r^ from pCF200 was exchanged to Neo^r^ from pMU131 (23) by Gibson Assembly. Afterward, based on pCF404, the Neo^r^ under the control of the P*_mcrB_*_(_*_M.v._*_)_ promoter and the T*_mcr_* terminator was used to construct a putative integration plasmid for the exchange of an annotated *pyrF* gene in *M. thermautotrophicus* ΔH, using AscI and FseI as restriction enzymes and T4 ligase for ligation, resulting in pCF407. In pCF407 the P*_mcrB_*_(_*_M.v._*_)_ promoter was exchanged to P_synth_ by inverse PCR resulting in pSB1. The fragments P*_mcrB_*_Neo^r^_T*_mcr_* from pCF407 and P_synth__Neo^r^_T*_mcr_* from pSB1 were used to substitute for the Sim^r^ in pSV1_2 by restriction-ligation cloning using AscI and FseI, resulting in pSV1_3 and pMVS-V1, respectively. To generate pMVS1111A:P_synth_-*bgaB*, the PCR-amplified gBlock with the thermostable β-galactosidase (*bgaB*) gene, which was codon optimized for *M. thermautotrophicus* ΔH and which was placed under the control of the P_synth_ promoter, was fused to AscI-digested pMVS-V1 with Gibson Assembly. The AscI restriction enzyme-recognition sequence was recovered at the intersection with the selectable-marker module, and a PacI sequence was introduced at the intersection with the *M. thermautotrophicus* ΔH replicon module. Further promoters [P_synth(BRE),_ P*_hmtB_*, P*_mrt_*_(_*_M.t._*_)_] were amplified via overlap-extension PCR of the β-galactosidase gBlock and promoter gBlock and inserted by restriction/ligation cloning using restriction enzymes PacI and AscI. pCF201 was constructed by amplifying the *fdh*_Z-245_ operon from *M. thermautotrophicus* Z-245 genomic DNA and introducing the fragment into pUC19 by Gibson Assembly. Gibson Assembly was used to exchange the Neo^r^-coding region in pCF204 with the *fdh*_Z-245_ operon from pCF201, resulting in plasmid pLM201. The promoter P*_mcrB_*_(_*_M.v._*_)_ in pLM201 was exchanged to P*_hmtB_* by inverse PCR of the complete plasmid, except of the P*_mcrB_*_(_*_M.v._*_)_ sequence, with primers containing overlapping parts of P*_hmtB_* in the overhangs, and direct transformation of E. coli with the linear PCR product. The resulting plasmid pLM202 was used to amplify the P*_hmtB_*-*fdh*_Z-245_ cassette by PCR to include PacI and AscI restriction enzyme-recognition sequences. Restriction/ligation cloning with the restriction enzymes PacI and AscI was used to exchange the β-galactosidase gene in pMVS1111A:P_synth_-*bgaB* for the P*_hmtB_*-*fdh*_Z-245_ cassette to give pMVS1111A:P*_hmtB_*-*fdh*_Z-245_.

### Plasmid DNA extraction from *Methanothermobacter* spp.

For plasmid DNA extraction from *Methanothermobacter* spp., 10 ml of liquid cell culture was centrifuged at 3,700 rpm for 15 min at room temperature (Centrifuge 5920 R, rotor S-4x1000; Eppendorf, Hamburg, Germany). The supernatant was discarded, and the cell pellet was resuspended in 150 μl of sucrose (30 wt%)-containing buffer P1 (from QIAprep Spin miniprep kit; Qiagen, Hilden, Germany). For lysis of *Methanothermobacter* cells, alkaline lysis was combined with enzymatic lysis by adding a final concentration of 100 ng/ml of pseudomurein endoisopeptidase (PeiP) to the sample prior to incubation for 1 h at 60°C. The pseudomurein-degrading enzyme PeiP, which lyses pseudomurein-containing *Methanobacteriales* cell walls, was produced as a heterologous 6×His-tagged protein from pME2508-carrying E. coli BL21(DE3) as described previously (48). The recombinant protein was purified via a Protino Ni-TED column according to the manufacturer’s guidelines (Macherey+Nagel, Düren, Germany). After the PeiP treatment, the QIAprep Spin miniprep kit (Qiagen, Hilden, Germany) manufacturer’s guidelines were followed with final elution in 40 μl nuclease-free water.

### Interdomain conjugational DNA transfer.

DNA transfer via interdomain conjugation was achieved between E. coli S17-1 and *M. thermautotrophicus* ΔH. E. coli S17-1 was transformed with the respective shuttle vector. Overnight cultures of the respective E. coli S17-1 donor strains were inoculated. At the same time, 20 ml of liquid mineral medium was inoculated with wild-type *M. thermautotrophicus* ΔH (recipient). The overnight culture of E. coli S17-1, which contained the shuttle vector, was diluted into 10 ml of fresh LB medium in a sterile 50-ml baffled flask for better aeration to give an OD_600_ of 0.3 to 0.5. When this culture reached an OD_600_ of 2.0 to 2.5, the incubation was stopped and the culture was harvested aerobically at 3,700 rpm for 10 min at room temperature (Centrifuge 5920 R, rotor S-4x1000; Eppendorf, Hamburg, Germany). The supernatant was discarded, and the E. coli S17-1 pellet was transferred into the anaerobic chamber. Wild-type *M. thermautotrophicus* ΔH was grown to early stationary growth phase (OD_600_ of 0.25 to 0.35). 8 ml of *M. thermautotrophicus* ΔH culture were centrifuged stepwise at 12,500 rpm for 4 min at room temperature (MySPIN 12 minicentrifuge; Thermo Scientific, Waltham, MA, USA) inside the anaerobic chamber. The final pellet was resuspended in 250 μl of the original nonconcentrated *M. thermautotrophicus* ΔH culture and gently mixed with the E. coli S17-1 pellet. 100 μl of cell suspension were anaerobically spotted on solid LB-MS medium, which was a mixture that consisted of 50 vol% of mineral medium and 50 vol% of LB medium without the 10 g/liter sodium chloride. The spot was dried, while the lid of the petri dish was kept slightly open for 1 h at 37°C in the incubator (Coy Laboratory Products, Grass Lake, MI, USA) within the anaerobic chamber. When the spot was completely absorbed, the plates were provided with paper clips and transferred to a stainless-steel jar. The gas phase of the jar was exchanged to 200 kPa H_2_/CO_2_/H_2_S (79.9/20/0.1 vol%) and incubated at 37°C without shaking for 16 to 20 h. The spot-mated E. coli S17-1 and *M. thermautotrophicus* ΔH cells were washed from the LB-MS plates using 1 ml nonselective mineral medium and transferred to 4 ml nonselective mineral medium in a 50-ml serum bottle with an H_2_/CO_2_ (80/20 vol%) gas phase. After recovery for 3 to 4 h at 60°C with shaking at 150 rpm, 1 ml of the culture was transferred to 20 ml selective liquid mineral medium in a 100-ml serum bottle and incubated at 60°C with shaking at 150 rpm. Growth of *M. thermautotrophicus* ΔH after 24 to 48 h of incubation indicated successful DNA transfer into *M. thermautotrophicus* ΔH, while growth only later than 48 h indicated the appearance of spontaneously neomycin-resistant *M. thermautotrophicus* ΔH cells (these cultures can be discarded as unsuccessful). Fifty microliters from this selective-enrichment culture was spread plated on selective solidified medium plates, and individual colonies were analyzed after 2 days of incubation at 60°C. A larger amount of individual colonies can be obtained by pour plating, but the analysis of spread-plated cells is easier from an experimental handling point of view (discussed below).

To determine the conjugation frequency, the following modifications to the standard protocol were made: (i) the cell count of *M. thermautotrophicus* ΔH in liquid culture was determined by counting in a Petroff counting chamber (the initial recipient cell number in 100 μl of the stepwise-concentrated culture was calculated based on this cell count), (ii) the 5-ml nonselective recovery culture from the washed spot after spot mating was incubated for ∼16 to 20 h instead of 3 to 4 h, and (iii) 100 μl of the nonselective recovery culture was directly spread plated on selective solidified medium plates, without a liquid selective-enrichment step.

### Molecular methods for analysis of genetically modified *M. thermautotrophicus* ΔH.

PCR analysis was performed from liquid cultures and directly from individual colonies. 100 μl of liquid culture or one individual colony, which was resuspended in 40 μl of deionized water, was boiled for 12 min at 100°C (ThermoMixer C; Eppendorf, Hamburg, Germany). After cooling the sample on ice, 1 μl was added to a 10-μl PCR mix. PCR was performed using Phire plant PCR master mix (Thermo Scientific, Waltham, MA, USA). The denaturation and annealing times were increased from 5 s to 20 s and to 10 s, respectively. Thirty cycles were performed for all analyses. We observed false-positive PCR signals for shuttle-vector DNA due to plasmid DNA carryover from E. coli for two transfers after the nonselective liquid recovery step. After the third transfer, plasmid DNA from E. coli was not detectable anymore in any of our experiments. For robust PCR amplifications of individual colonies from *M. thermautotrophicus* ΔH, it was crucial to keep the agar contamination of the PCR sample as low as possible. Therefore, even though the plating efficiency is higher with pour plating, genetically modified *M. thermautotrophicus* ΔH strains were spread plated instead of pour plated. This led to a lower total colony count but to more reliable results.

Additional to PCR analysis, plasmid DNA from genetically modified *M. thermautotrophicus* ΔH strains was extracted as described above. The purified plasmid DNA was used for retransformation of E. coli NEB stable. Analysis of E. coli NEB stable colonies was performed via test restriction digestions and Sanger sequencing for further confirmation of stable replication of shuttle vectors in *M. thermautotrophicus* ΔH.

### β-Galactosidase enzyme activity assays.

For a qualitative β-galactosidase enzyme activity assay with the lactose analogue S-Gal, 2 ml of overnight cell cultures that carry pMVS-V1 or pMVS1111A:P_Synth_-*bgaB* was harvested by centrifugation for 4 min at 13,000 rpm at room temperature (Centrifuge 5424, rotor FA-45-24-11; Eppendorf, Hamburg, Germany). The supernatant was discarded, and the samples were stored at −20°C until further use. All samples were resuspended in 100 μl buffer P1 (from Qiagen QIAprep Spin miniprep kit) containing sucrose (30 wt%) and lysed by adding 100 ng/ml PeiP, followed by incubation for 30 min at 60°C. Fifty microliters of the cell lysate was incubated with 250 μg/ml S-Gal and 250 μg/ml ammonium ferric citrate in 1 ml LB medium, which provided any potentially required trace compounds. The samples were incubated for 1 h at 60°C. After ∼30 min, a color change was visible.

For a quantitative β-galactosidase enzyme activity assay with the lactose analogue ONPG, 4 ml of cell culture was harvested anaerobically by stepwise centrifugation (Centrifuge 5424, rotor FA-45-24-11; Eppendorf, Hamburg, Germany). Afterward, the same lysis procedure for samples was applied as for the S-Gal assay. The resulting cell lysate was used for a quantitative *in vitro* β-galactosidase enzyme activity assay with ONPG as chromogenic substance according to the method of Jensen et al. (31). In brief, 12.5 μl of cell lysate (equal to 0.5 ml of original cell culture) was mixed with 600 μl of ONPG (1 mg/ml)-containing substrate solution. The mixture was incubated for 2 h at 60°C. Afterward, 200 μl was added to 200 μl of 1 M sodium bicarbonate stop solution in a 96-well plate. The absorbance at 420 nm was measured in a microplate reader (Multiskan Go; Thermo Scientific, Waltham, MA, USA). For the preliminary experiment (see Fig. S7 in the supplemental material), 25 μl of cell lysate was mixed with 675 μl of ONPG substrate solution instead. After the incubation for 2 h at 60°C, 350 μl of substrate solution was added to 350 μl of stop solution, and the absorbance at 420 nm was measured in a cuvette (1-cm path length) with a spectrophotometer (NP80; Implen, Munich, Germany). Enzyme activity was defined in Miller units as change in absorbance at 420 nm per assay time in hours, optical density at 600 nm, and volume of *M. thermautotrophicus* ΔH cell culture [Δ*A*_420_ × (h × OD_600_ × liter)^−1^].

10.1128/mBio.02766-21.8 FIG S7 (A to D) The measurements of absorbance at 420 nm (A_420_) of the enzyme activity assay (bars) and optical density at 600 nm (OD_600_) of the corresponding genetically engineered *M. thermautotrophicus* ΔH strain (dots) after different incubation periods [*M. thermautotrophicus* ΔH with pMVS-V1 (A), pMVS1111A:P*_mrt_*_(_*_M.t_*_.)_-*bgaB* (B), pMVS1111A:P_synth_-*bgaB* (C), or pMVS1111A:P*_hmtB_*-*bgaB* (D)]. (E) The resulting Miller units from panels A to D for the four different *M. thermautotrophicus* ΔH strains are given for the incubation periods 15 h, 19 h, 24 h, and 36 h for each strain from left to right. Average [pMVS-V1 (*n *= 2), pMVS1111A:P*_mrt_*_(_*_M.t_*_.)_-*bgaB* (*n *= 3), pMVS1111A:P_synth_-*bgaB* (*n *= 2), and pMVS1111A:P*_hmtB_*-*bgaB* (*n *= 3)] with error bars indicating standard deviation. The Miller units in this experiment are lower than in the experiment in Fig. 3C because of differences in the experimental parameters (Materials and Methods). Download FIG S7, EPS file, 1.9 MB. Copyright © 2021 Fink et al. 2021 Fink et al. https://creativecommons.org/licenses/by/4.0/ This content is distributed under the terms of the Creative Commons Attribution 4.0 International license.

### High-performance liquid chromatography for formate analysis.

Formate concentrations were analyzed via a high-pressure liquid chromatography (HPLC) system as described in the work of Klask et al. (49). For HPLC sample preparation, all culture samples were centrifuged for 5 min at 13,000 rpm (Centrifuge 5424; Eppendorf, Germany) in 2-ml reaction tubes. 450 μl of the supernatant were transferred into clean reaction tubes and stored at −20°C until use. Frozen samples were thawed at room temperature. The samples were vortexed and centrifuged again, and 400 μl of the supernatant was transferred into short-thread HPLC/gas chromatography (GC) vials (glass vial ND9; VWR, Germany) and sealed with short screw caps, which contained rubber septa (6 mm for ND9; VWR, Germany). Formate standards (1 to 500 μM) were prepared freshly for the analysis. All HPLC samples were randomized.

## References

[1] Thauer RK, Kaster AK, Seedorf H, Buckel W, Hedderich R. 2008. Methanogenic archaea: ecologically relevant differences in energy conservation. Nat Rev Microbiol 6:579–591. doi:10.1038/nrmicro1931. 18587410

[2] Thauer RK. 1998. Biochemistry of methanogenesis: a tribute to Marjory Stephenson: 1998 Marjory Stephenson prize lecture. Microbiology 144:2377–2406. doi:10.1099/00221287-144-9-2377. 9782487

[3] Thauer RK. 2015. My lifelong passion for biochemistry and anaerobic microorganisms. Annu Rev Microbiol 69:1–30. doi:10.1146/annurev-micro-091014-104344. 26488272

[4] Kaster A-K, Goenrich M, Seedorf H, Liesegang H, Wollherr A, Gottschalk G, Thauer RK. 2011. More than 200 genes required for methane formation from H_2_ and CO_2_ and energy conservation are present in *Methanothermobacter marburgensis* and *Methanothermobacter thermautotrophicus*. Archaea 2011:973848. doi:10.1155/2011/973848. 21559116 PMC3087415

[5] Thauer RK. 2019. Methyl (alkyl)-coenzyme M reductases: nickel F-430-containing enzymes involved in anaerobic methane formation and in anaerobic oxidation of methane or of short chain alkanes. Biochemistry 58:5198–5220. doi:10.1021/acs.biochem.9b00164. 30951290 PMC6941323

[6] Pende N, Sogues A, Megrian D, Sartori-Rupp A, England P, Palabikyan H, Rittmann SK-MR, Graña M, Wehenkel AM, Alzari PM, Gribaldo S. 2021. SepF is the FtsZ anchor in archaea, with features of an ancestral cell division system. Nat Commun 12:3214. doi:10.1038/s41467-021-23099-8. 34088904 PMC8178401

[7] Kandler O, König H. 1998. Cell wall polymers in archaea (archaebacteria). Cell Mol Life Sci 54:305–308. doi:10.1007/s000180050156. 9614965 PMC11147200

[8] Thema M, Weidlich T, Hörl M, Bellack A, Mörs F, Hackl F, Kohlmayer M, Gleich J, Stabenau C, Trabold T, Neubert M, Ortloff F, Brotsack R, Schmack D, Huber H, Hafenbradl D, Karl J, Sterner M. 2019. Biological CO_2_-methanation: an approach to standardization. Energies 12:1670. doi:10.3390/en12091670.

[9] Pfeifer K, Ergal İ, Koller M, Basen M, Schuster B, Rittmann SK-MR. 2021. Archaea biotechnology. Biotechnol Adv 47:107668. doi:10.1016/j.biotechadv.2020.107668. 33271237

[10] Martin MR, Fornero JJ, Stark R, Mets L, Angenent LT. 2013. A single-culture bioprocess of *Methanothermobacter thermautotrophicus* to upgrade digester biogas by CO_2_-to-CH_4_ conversion with H_2_. Archaea 2013:157529. doi:10.1155/2013/157529. 24194675 PMC3806361

[11] Seifert AH, Rittmann S, Herwig C. 2014. Analysis of process related factors to increase volumetric productivity and quality of biomethane with *Methanothermobacter marburgensis*. Appl Energy 132:155–162. doi:10.1016/j.apenergy.2014.07.002.

[12] Meile L, Reeve JN. 1985. Potential shuttle vectors based on the methanogen plasmid pME2001. Nat Biotechnol 3:69–72. doi:10.1038/nbt0185-69.

[13] Leisinger T, Meile L. 1993. Plasmids, phages, and gene transfer in methanogenic bacteria, p 1–12. *In* Sebald M (ed), Genetics and molecular biology of anaerobic bacteria. Springer, New York, NY.

[14] Luo Y, Leisinger T, Wasserfallen A. 2001. Comparative sequence analysis of plasmids pME2001 and pME2200 of *Methanothermobacter marburgensis* strains Marburg and ZH3. Plasmid 45:18–30. doi:10.1006/plas.2000.1493. 11319928

[15] Meile L, Abendschein P, Leisinger T. 1990. Transduction in the archaebacterium *Methanobacterium thermoautotrophicum* Marburg. J Bacteriol 172:3507–3508. doi:10.1128/jb.172.6.3507-3508.1990. 2345156 PMC209168

[16] Worrell VE, Nagle DP, McCarthy D, Eisenbraun A. 1988. Genetic transformation system in the archaebacterium *Methanobacterium thermoautotrophicum* Marburg. J Bacteriol 170:653–656. doi:10.1128/jb.170.2.653-656.1988. 3422229 PMC210704

[17] Jenal U, Rechsteiner T, Tan PY, Bühlmann E, Meile L, Leisinger T. 1991. Isoleucyl-tRNA synthetase of *Methanobacterium thermoautotrophicum* Marburg. Cloning of the gene, nucleotide sequence, and localization of a base change conferring resistance to pseudomonic acid. J Biol Chem 266:10570–10577. doi:10.1016/S0021-9258(18)99261-6. 2037598

[18] Meile L, Stettler R, Banholzer R, Kotik M, Leisinger T. 1991. Tryptophan gene cluster of *Methanobacterium thermoautotrophicum* Marburg: molecular cloning and nucleotide sequence of a putative *trpEGCFBAD* operon. J Bacteriol 173:5017–5023. doi:10.1128/jb.173.16.5017-5023.1991. 1860817 PMC208190

[19] Majernik A, Cubonova L, Polak P, Smigan P, Greksak M. 2003. Biochemical analysis of neomycin-resistance in the methanoarchaeon *Methanothermobacter thermautotrophicus* and some implications for energetic processes in this strain. Anaerobe 9:31–38. doi:10.1016/S1075-9964(03)00042-8. 16887685

[20] Rospert S, Linder D, Ellermann J, Thauer RK. 1990. Two genetically distinct methyl‐coenzyme M reductases in *Methanobacterium thermoautotrophicum* strain Marburg and ΔH. Eur J Biochem 194:871–877. doi:10.1111/j.1432-1033.1990.tb19481.x. 2269306

[21] Waege I, Schmid G, Thumann S, Thomm M, Hausner W. 2010. Shuttle vector-based transformation system for *Pyrococcus furiosus*. Appl Environ Microbiol 76:3308–3313. doi:10.1128/AEM.01951-09. 20363792 PMC2869139

[22] Santangelo TJ, Cubonová L, Matsumi R, Atomi H, Imanaka T, Reeve JN. 2008. Polarity in archaeal operon transcription in *Thermococcus kodakaraensis*. J Bacteriol 190:2244–2248. doi:10.1128/JB.01811-07. 18192385 PMC2258858

[23] Shaw AJ, Hogsett DA, Lynd LR. 2010. Natural competence in *Thermoanaerobacter* and *Thermoanaerobacterium* species. Appl Environ Microbiol 76:4713–4719. doi:10.1128/AEM.00402-10. 20472726 PMC2901744

[24] Susanti D, Frazier MC, Mukhopadhyay B. 2019. A genetic system for *Methanocaldococcus jannaschii:* an evolutionary deeply rooted hyperthermophilic methanarchaeon. Front Microbiol 10:1256. doi:10.3389/fmicb.2019.01256. 31333590 PMC6616113

[25] Fonseca DR, Halim MFA, Holten MP, Costa KC. 2020. Type IV-like pili facilitate transformation in naturally competent archaea. J Bacteriol 202:e00355-20. doi:10.1128/JB.00355-20. 32817089 PMC7549367

[26] Martinez-Garcia E, Aparicio T, Goni-Moreno A, Fraile S, de Lorenzo V. 2015. SEVA 2.0: an update of the Standard European Vector Architecture for de-/re-construction of bacterial functionalities. Nucleic Acids Res 43:D1183–D1189. doi:10.1093/nar/gku1114. 25392407 PMC4383931

[27] Heap JT, Pennington OJ, Cartman ST, Minton NP. 2009. A modular system for *Clostridium* shuttle plasmids. J Microbiol Methods 78:79–85. doi:10.1016/j.mimet.2009.05.004. 19445976

[28] Hoseki J, Yano T, Koyama Y, Kuramitsu S, Kagamiyama H. 1999. Directed evolution of thermostable kanamycin-resistance gene: a convenient selection marker for *Thermus thermophilus*. J Biochem 126:951–956. doi:10.1093/oxfordjournals.jbchem.a022539. 10544290

[29] Molitor B, Kirchner K, Henrich AW, Schmitz S, Rosenbaum MA. 2016. Expanding the molecular toolkit for the homoacetogen *Clostridium ljungdahlii*. Sci Rep 6:31518. doi:10.1038/srep31518. 27527841 PMC4985741

[30] Dodsworth JA, Li L, Wei S, Hedlund BP, Leigh JA, de Figueiredo P. 2010. Interdomain conjugal transfer of DNA from bacteria to archaea. Appl Environ Microbiol 76:5644–5647. doi:10.1128/AEM.00967-10. 20581182 PMC2918978

[31] Jensen TO, Pogrebnyakov I, Falkenberg KB, Redl S, Nielsen AT. 2017. Application of the thermostable β-galactosidase, BgaB, from *Geobacillus stearothermophilus* as a versatile reporter under anaerobic and aerobic conditions. AMB Express 7:169–179. doi:10.1186/s13568-017-0469-z. 28875485 PMC5585113

[32] Darcy TJ, Hausner W, Awery DE, Edwards AM, Thomm M, Reeve JN. 1999. *Methanobacterium thermoautotrophicum* RNA polymerase and transcription *in vitro*. J Bacteriol 181:4424–4429. doi:10.1128/JB.181.14.4424-4429.1999. 10400604 PMC93948

[33] Wood GE, Haydock AK, Leigh JA. 2003. Function and regulation of the formate dehydrogenase genes of the methanogenic archaeon *Methanococcus maripaludis*. J Bacteriol 185:2548–2554. doi:10.1128/JB.185.8.2548-2554.2003. 12670979 PMC152622

[34] Wasserfallen A, Nölling J, Pfister P, Reeve J, De Macario EC. 2000. Phylogenetic analysis of 18 thermophilic *Methanobacterium* isolates supports the proposals to create a new genus, *Methanothermobacter* gen. nov., and to reclassify several isolates in three species, *Methanothermobacter thermautotrophicus* comb. nov., *Methanothermobacter wolfeii* comb. nov., and *Methanothermobacter marburgensis* sp. nov. Int J Syst Evol Microbiol 50:43–53. doi:10.1099/00207713-50-1-43. 10826786

[35] Nölling J, Reeve JN. 1997. Growth-and substrate-dependent transcription of the formate dehydrogenase (*fdhCAB*) operon in *Methanobacterium thermoformicicum* Z-245. J Bacteriol 179:899–908. doi:10.1128/jb.179.3.899-908.1997. 9006048 PMC178775

[36] Argyle JL, Tumbula DL, Leigh JA. 1996. Neomycin resistance as a selectable marker in *Methanococcus maripaludis*. Appl Environ Microbiol 62:4233–4237. doi:10.1128/aem.62.11.4233-4237.1996. 8900017 PMC168247

[37] Jones WJ, Whitman WB, Fields RD, Wolfe RS. 1983. Growth and plating efficiency of *Methanococci* on agar media. Appl Environ Microbiol 46:220–226. doi:10.1128/aem.46.1.220-226.1983. 16346342 PMC239291

[38] Ao X, Li Y, Wang F, Feng M, Lin Y, Zhao S, Liang Y, Peng N. 2013. The *Sulfolobus* initiator element is an important contributor to promoter strength. J Bacteriol 195:5216–5222. doi:10.1128/JB.00768-13. 24039266 PMC3811598

[39] Gregor D, Pfeifer F. 2005. *In vivo* analyses of constitutive and regulated promoters in halophilic archaea. Microbiology (Reading) 151:25–33. doi:10.1099/mic.0.27541-0. 15632422

[40] Peng N, Xia Q, Chen Z, Liang YX, She Q. 2009. An upstream activation element exerting differential transcriptional activation on an archaeal promoter. Mol Microbiol 74:928–939. doi:10.1111/j.1365-2958.2009.06908.x. 19818017

[41] Enzmann F, Mayer F, Rother M, Holtmann D. 2018. Methanogens: biochemical background and biotechnological applications. AMB Express 8:1. doi:10.1186/s13568-017-0531-x. 29302756 PMC5754280

[42] Nayak DD, Metcalf WW. 2017. Cas9-mediated genome editing in the methanogenic archaeon *Methanosarcina acetivorans*. Proc Natl Acad Sci USA 114:2976–2981. doi:10.1073/pnas.1618596114. 28265068 PMC5358397

[43] Lyu Z, Jain R, Smith P, Fetchko T, Yan Y, Whitman WB. 2016. Engineering the autotroph *Methanococcus maripaludis* for geraniol production. ACS Synth Biol 5:577–581. doi:10.1021/acssynbio.5b00267. 26886063

[44] Balch W, Fox G, Magrum L, Woese C, Wolfe R. 1979. Methanogens: reevaluation of a unique biological group. Microbiol Rev 43:260–296. doi:10.1128/mr.43.2.260-296.1979. 390357 PMC281474

[45] Pozzi R, Coles M, Linke D, Kulik A, Nega M, Wohlleben W, Stegmann E. 2016. Distinct mechanisms contribute to immunity in the lantibiotic NAI-107 producer strain *Microbispora* ATCC PTA-5024. Environ Microbiol 18:118–132. doi:10.1111/1462-2920.12892. 25923468

[46] Sambrook J, Fritsch EF, Maniatis T. 1989. Molecular cloning: a laboratory manual. Cold Spring Harbor Laboratory Press, Cold Spring Harbor, NY.

[47] Sarmiento F, Leigh JA, Whitman WB. 2011. Genetic systems for hydrogenotrophic methanogens. Methods Enzymol 494:43–73. doi:10.1016/B978-0-12-385112-3.00003-2. 21402209

[48] Luo Y, Pfister P, Leisinger T, Wasserfallen A. 2002. Pseudomurein endoisopeptidases PeiW and PeiP, two moderately related members of a novel family of proteases produced in *Methanothermobacter* strains. FEMS Microbiol Lett 208:47–51. doi:10.1111/j.1574-6968.2002.tb11059.x. 11934493

[49] Klask C-M, Kliem-Kuster N, Molitor B, Angenent LT. 2020. Nitrate feed improves growth and ethanol production of *Clostridium ljungdahlii* with CO_2_ and H_2_, but results in stochastic inhibition events. Front Microbiol 11:724. doi:10.3389/fmicb.2020.00724. 32435236 PMC7219301

[50] Bokranz M, Klein A, Meile L. 1990. Complete nucleotide sequence of plasmid pME2001 of *Methanobacterium thermoautotrophicum* (Marburg). Nucleic Acids Res 18:363. doi:10.1093/nar/18.2.363. 2326168 PMC330278

[51] Kovach ME, Elzer PH, Steven Hill D, Robertson GT, Farris MA, Roop RM, Peterson KM. 1995. Four new derivatives of the broad-host-range cloning vector pBBR1MCS, carrying different antibiotic-resistance cassettes. Gene 166:175–176. doi:10.1016/0378-1119(95)00584-1. 8529885

[52] Yanisch-Perron C, Vieira J, Messing J. 1985. Improved M13 phage cloning vectors and host strains: nucleotide sequences of the M13mpl8 and pUC19 vectors. Gene 33:103–119. doi:10.1016/0378-1119(85)90120-9. 2985470

