# Supplementary material for: A Shuttle-Vector System Allows Heterologous Gene Expression in the Thermophilic Methanogen Methanothermobacter thermautotrophicus ΔH
Source: mBio. 2021 Nov 23;12(6):e02766-21. doi: 10.1128/mBio.02766-21 (PMC8609365; doi:10.1128/mBio.02766-21)
Supplement: TEXT S1 [file mbio.02766-21-t0001.docx]

**Supplementary Text S1**

**Supplementary Results**

**Supplementary Text S1A – Plating efficiencies**

Growth on solidified media plates was investigated with spot-, spread-, and pour-plating (**Materials and Methods**). With spot-plating, colonies are barely distinguishable, but concentrated and dense growth can be obtained (**Figure S1A**). With spread- and pour-plating, we obtained individual colonies (**Figure S1B, C**), and therefore we further investigated factors that influence plating efficiencies with these techniques. While the experimental variance was high in individual experiments and strongly depended on many different factors, as further discussed below, some factors had a distinct impact on the plating efficiency. After we had optimized the plating conditions, we performed a set of experiments to compare the plating efficiency with as little experimental variance as possible (**Figure S1D**). The addition of 0.1 volume% hydrogen sulfide in the headspace gas mixture, as additional reducing agent and sulfur source, resulted in an increase of the number of individual colonies by one order of magnitude when compared to the same plating procedure without hydrogen sulfide gas (**Figure S1D**). With hydrogen sulfide gas and with spread-plating of cells in the stationary growth phase, the plating-efficiency was found to be 1.2±0.5% (*n*=6; **Figure S1D**), while with spread-plating of cells in the exponential growth phase, a plating efficiency of 5±2% (*n*=4) was reached (**Figure S1D**). When we used pour-plating with the same number of cells in the stationary growth phase as with spread-plating, and with hydrogen sulfide gas, the number of individual colonies increased drastically by two orders of magnitude with a plating efficiency of up to 50% (**Figure S3B**) or higher (135±10%, *n*=3; **Figure S1D**), whereas the colony size decreased when compared to colonies from spread-plating (**Figure S1B, C**). However, larger colonies on top of the pour-plated solidified media plates were also observed (**Figure S1B**). Plating efficiencies of more than 100% and high standard deviations in independent experiments appeared from incomplete counting, because of the formation of filaments or clumps of cells, and from a degree of sensitivity of the plating procedure to variations in the experimental handling, such as differences in the exact growth phase of the plated culture or mineral media batches (**Supplementary** **Text S1J**). In many experiments, we realized that the plating efficiency decreased considerably, when water accumulated inside the plate, and formed a layer on the side of the plate, which led to a seal between the bottom of the petri dish-plate and the lid and prevented sufficient gas exchange. As a protective measure, we implemented the addition of paper clips on the edges of the plate to lift up the lid slightly, which efficiently prevented water from sealing the plates (**Figure S1E; Supplementary Text S1J**).

**Supplementary Text S1B – Growth-inhibiting effects of antibiotics**

We investigated the growth-inhibiting effects of common antibiotics (simvastatin/mevilonin, neomycin, and puromycin) on *M. thermautotrophicus* ΔH in liquid cultures, which are known to inhibit the growth of methanogens ^1-3^. We found that non-selective conditions result in a densely grown culture within 24 h. For simvastatin, we tested concentrations ranging from 0-21.5 µg/mL (0-50 µM) on ~5·10^5^ cells/mL, and found that 13 µg/mL (30 µM) inhibited growth at least for 48 h. We also tested the inhibition by the simvastatin-analog mevilonin, but did not find a growth-inhibiting effect on *M. thermautotrophicus* ΔH up to a concentration of 21.5 µg/mL (50 µM). For neomycin, we tested concentrations ranging from 0-250 µg/mL on ~5·10^5^ cells/mL initial cell density. We found that 50 µg/mL inhibited growth for less than 24 h, 100 µg/mL inhibited growth for at least 48 h, and 250 µg/ml neomycin for at least 60 h of incubation, while we did not analyze the growth-inhibiting effect beyond an incubation period of 60 h (**Figure S2**). We further investigated puromycin in liquid mineral medium, because this antibiotic is commonly used in genetic systems for mesophilic methanogens ^1,4^, and we found good inhibition from 50 µg/mL on ~5·10^5^ cells/mL initial cell density for at least 72 h. However, the available selectable marker has not yet been adapted to thermophilic conditions to confer resistance against puromycin at elevated temperature. Thus, we did not proceed with this antibiotic.

With having the plating efficiency defined (**Supplementary** **Text S1A**), we further investigated antibiotics for the inhibitory effects on solidified media plates. For simvastatin, we tested concentrations ranging from 0-21.5 µg/mL (0-50 µM) on the inhibition of 1·10^8^ cells with pour-plating (**Figure S3A**). While up to 8.7 µg/mL (20 µM) simvastatin resulted in a microbial lawn of *M. thermautotrophicus* ΔH cells, inhibition was recognizable from 13 µg/mL (30 µM) upwards. At 21.5 µg/mL (50 µM) we observed only 27±10 (*n*=3) individual colonies after an incubation period of 48 h (**Figure S3A**), which corresponds to a growth inhibitory efficiency of close to 100%. For neomycin, we tested concentrations ranging from 0-250 µg/mL on the inhibition of 1·10^3^ cells with pour-plating (**Figure S3B**). At 50 µg/mL no significant reduction of the number of colonies was observed when compared to non-selective conditions for which we achieved a plating efficiency of ~50% (**Figure S3B**). With 100 µg/mL only 30±5 (*n*=3) individual colonies, and with 250 µg/mL only 3±1 (*n*=3) individual colonies were observed after an incubation period of 48 h, respectively (**Figure S3B**). Therefore, we conclude that 250 µg/mL of neomycin inhibits growth of wild-type *M. thermautotrophicus* ΔH on solidified media plates with an efficiency of >99.9% for at least 48 h (**Figure S3B**). We further tested a genetically modified *M. thermautotrophicus* ΔH strain, which carries pMVS-V1 (**Figure 1**), under selective conditions in liquid mineral medium and on solidified media plates. We found that pMVS-V1 relieves the growth-inhibiting effect of 250 µg/mL neomycin, and resulted in wild-type-like growth (**Figure S2 and S3B**).

**Supplementary Text S1C – pMVS design**

The five modules of the pMVS design are separated by rare eight base-pair recognition sequences as described in the main manuscript (*Pme*I, *AsiS*I, *Fse*I, *Asc*I, and *Pac*I). The archetype shuttle vector for this design is pMVS-V1, which does not contain a *Pac*I site (**Figure 1**). We selected the pMTL83151 plasmid from the pMTL80000 system ^5^ as the source for the backbone for *E. coli* in pMVS-V1, because this plasmid contains the *tra*-region from RK2 (for mobilization of the plasmid) in addition to the ColE1 replicon. Furthermore, this plasmid already brings a chloramphenicol-selectable marker (Cam^r^) for selection in *E. coli*, which is separated from the replicon by a *Pme*I-recognition sequence, due to the modularity of the pMTL80000 system ^5^. As the replicon for *M. thermautotrophicus* ΔH, we chose the cryptic plasmid pME2001 from *M. marburgensis* ^6^. This plasmid has been studied to some extent and is the smallest plasmid, which is known in *Methanothermobacter* spp. ^7-9^. The plasmid contains five open-reading frames (*orf1-5*) with barely annotated functions. Additionally, in a ~1‑kilobase section on pME2001, no open-reading frames have been annotated, but instead this section contains five sites of inserted fragments (IFs), which are different in pME2001 when compared to the similar cryptic plasmid pME2200 from *M. thermautotrophicus* ZH3, whereas IF5 only occurs in pME2001 ^7^. Previous attempts to create *E. coli*-*M. thermautotrophicus* shuttle vectors relied on the availability of restriction enzyme-recognition sequences in pME2001 ^8^. To not interrupt one of the open-reading frames and simultaneously to not intersect the region of a potential origin of replication for which the exact location is not known in pME2001 and pME2200, we decided to fuse the pME2001 replicon to the other components of the shuttle vector at the position of IF5 *via* Gibson^®^ Assembly (**Materials and Methods**), and to use the entire plasmid pME2001 as the replicon for *M. thermautotrophicus* ΔH.

We chose the thermostable neomycin-selectable marker (Neo^r^) from pMU131 for positive selection in *M. thermautotrophicus* ΔH ^10^. As a promoter, we selected a sequence published by Santangelo, et al. ^11^, which we designate P_synth_ (but which is called P*_hmtB_* in Santangelo, et al. ^11^, **Figure 3**), because the commonly used promoter P*_mcrB_*_(_*_M.v._*_)_ from *M. voltae* ^12^ did not result in genetically modified *M. thermautotrophicus* ΔH in combination with Neo^r^ in our hands. The promoter sequence from Santangelo, et al. ^11^ has similarity to the upstream region of a histone-binding protein (HmtB)-encoding gene from *M. thermautotrophicus* ΔH. However, several modifications had been introduced in P_synth_ compared to the native P*_hmtB_* sequence ^13^. The native P*_hmtB_* sequence was demonstrated to initiate *in-vitro* transcription, and thus, the generation of mRNA when using purified native *M. thermautotrophicus* ΔH RNA polymerase ^14^. Additionally, the P_synth_ promoter was shown to initiate gene expression in *T. kodakarensis* ^11^. As the terminator sequence for the selectable marker, we implemented the T*_mcr_* terminator sequence of the methyl-coenzyme M reductase (*mcr*) operon from *M. voltae*, which is commonly used in constructs for genetic modification of *Methanococcus* spp. and *Methanosarcina* spp. ^1^. We selected a thermostable β-galactosidase reporter (**Figure 3**) as our first gene of interest for the application module. When we introduced the thermostable β-galactosidase-encoding gene to pMVS-V1, which resulted in the shuttle vector pMVS1111A:P_synth_-*bgaB* with five modules, we included a *Pac*I site in addition, to complete the application module. The directionality of the *bgaB* gene was such that the T*_mcr_* terminator sequence from the selectable marker module is also terminating transcription of the *bgaB* gene (**Figure 1B;** **Materials and Methods**).

**Supplementary Text S1D – Analysis of successful DNA transfer *via* site-specific PCR**

We analyzed genetically modified *M. thermautotrophicus* ΔH strains *via* site-specific PCR amplifications with: **1)** a primer combination (**Table 1**), which specifically amplifies a 1-kilobase fragment of the pME2001 replicon; and **2)** primer combinations (**Table 1**), which specifically amplify either a 1.5-kilobase or a 2.8-kilobase fragment of genomic DNA from *M. thermautotrophicus* ΔH to confirm the integrity of the genetically modified strains (**Figure 2C;** **Materials and Methods**). In preliminary experiments with high densities of *E. coli* S17-1, we found that PCR signals for the presence of our high-copy number shuttle vectors (with regards to copy number in *E. coli*) could be obtained after up to three transfers in liquid media, and also from areas of solidified media plates at which no growth was observed, after up to two transfers. Therefore, to gather reliable results on the stable replication of shuttle vectors in *M. thermautotrophicus* ΔH, and to exclude false positive results from residual *E. coli* DNA, we first transferred cell material from individual colonies of putative *M. thermautotrophicus* ΔH transconjugants into selective liquid mineral medium. This enrichment culture was plated by streaking some culture with an inoculation loop on selective solidified media plates. From these plates, we inoculated another selective liquid mineral medium with cell material from individual colonies, and these liquid enrichment cultures were analyzed by PCR amplification. Successful DNA transfer into *M. thermautotrophicus* ΔH was reproducibly confirmed (**Figure 2C**).

**Supplementary Text S1E – Retransformation of *E. coli* with plasmid extracts from *M. thermautotrophicus* ΔH**

As a second approach to confirm the presence and integrity of the shuttle vector in *M. thermautotrophicus* ΔH, we extracted plasmid DNA from genetically modified *M. thermautotrophicus* ΔH cultures, and used this plasmid DNA for retransformation of *E. coli* NEB stable (**Materials and Methods**). Individual colonies of *E. coli* NEB stable were analyzed with restriction-enzyme digestion and Sanger sequencing. We found that all analyzed colonies contained the entire shuttle vector as deduced from the correct fragment sizes in the restriction-enzyme digestion (**Figure S4A**), and no mutations in the *M. thermautotrophicus* ΔH replicon as well as the neomycin-selectable marker (**Figure S4B**). We did not re-sequence the entire *E. coli* replicon and selectable marker (**Figure S4B**).

**Supplementary Text S1F – Conjugation frequency**

The determination of the conjugation frequency, which we define as transconjugants per initial recipient cells, is accompanied by several unknown parameters, such as the different incubation periods during the spot-mating, and the non-selective-recovery and selective-enrichments steps, before the selective spread-plating step to obtain individual colonies (**Figure 3**). Our standard protocol (**Materials and Methods**) reliably resulted in a high number of individual colonies, which were derived from an unknown number of transconjugants, because of the selective-enrichment step. Therefore, to quantify the conjugation frequency more accurately, we performed a subset of two independent experiments each in triplicate in which we did not include the selective-enrichment step, but instead we prolonged the incubation period for the non-selective-recovery step. These experiments resulted in 5±4 (*n*=6) colonies, with one colony in the lowest and ten colonies in the highest case. Still we had to take different parameters into account, and we calculated the conjugation frequency according to **Equation S1**:

$f= \frac{N_{P} \cdot V_{R}}{E \cdot N_{0} \cdot R_{W} \cdot2^{\left( D_{S}+D_{R} \right)}}$ **(Equation S1)**

where **f** represents the conjugation frequency; **N_P_** represents the number of individual colonies obtained after the final selective spread-plating step; **V_R_** represents the dilution factor for the amount of plated cells in relation to overall culture volume; **E** represents the spread-plating efficiency as a fraction; **N_0_** represents the initial recipient cell number used for spot-mating; **R_W_** represents the fraction of cells that are recovered from the washing step after the spot-mating; **D_S_** represents the number of cell divisions during spot-mating; and **D_R_** represents the number of cell divisions during non-selective recovery.

Because of the uncertainties accompanied with the experimental steps, we calculated a range of parameters in a simple sensitivity analysis assuming the worst and best case scenarios (**Table S1**). The parameters **V_R_**, **N_P_**, and **N_0_** are either known (dilution factor **V_R_** is 50 in our experiments, **Materials and Methods**), or can be experimentally determined by cell counting before the conjugational DNA transfer (**N_0_**) and colony counting of transconjugants after the selective spread-plating step (**N_P_**). The parameter **E** can be experimentally defined with a relatively good certainty, based on our plating efficiencies (**Figure S1**). However, the parameters **R_W_**, **D_S_**, and **D_R_** need to be estimated in a certain range for the sensitivity analysis, for example, based on assumptions that are made from other experiments. For **D_S_** this is because in the spot-mating step, *E. coli* and *M. thermautotrophicus* ΔH cells are combined and potential growth (number of cell divisions) of *M. thermautotrophicus* ΔH at 37°C cannot be determined. For our estimations, we define **D_S_** as either 0 or 1 cell divisions. For **R_W_**, the washing step after the spot-mating step is critical. While with careful suspension of the entire spot a recovery of all cells can be assumed, for our estimations, we define **R_W_** between 0.5 and 1.0 (for 50-100% recovery of cells). For **D_R_**, the overall number of cell divisions during the non-selective recovery step is critical. Based on typical growth experiments similar to these conditions (16-20 h without selection), we assume **D_R_** to be between 5 and 8 cell divisions. These assumptions result in an estimated conjugation frequency between 6·10^-6^ and 4·10^-9^ (**Table S1**).

**Supplementary Text S1G – Segregational stability of shuttle vector under non-selective conditions**

The segregational stability of shuttle vectors in *M. thermautotrophicus* ΔH is of interest for incubation periods under non-selective growth conditions (*e.g.*, in bioreactors), but also for potential plasmid-curing requirements. Therefore, we performed an experiment to assess the segregational stability of pMVS-V1. We inoculated both selective and non-selective liquid mineral medium with ~5·10^5^ cells/mL from a selectively grown pre-culture of pMVS-V1-carrying *M. thermautotrophicus* ΔH. After growth to ~1·10^8^ cells/mL, which corresponds to ~7-8 cell divisions (**Equation S2**), we repeated the transfer of ~5·10^5^ cells/mL from the non-selective liquid mineral medium to non-selective conditions twice. This resulted, in total, in ~21-28 cell divisions under non-selective conditions. We calculated the number of cell divisions according to **Equation S2**:

$n= \frac{log\left( N_{t} \right)-log(N_{0})}{log(2)}$ **(Equation S2)**

where **n** is the number of generations (cell divisions), **N_t_** is the cell concentration at the end of the incubation period, and **N_0_** is the cell concentration in the beginning of the incubation period.

We spread-plated cells from the first selective transfer to selective solidified media plates, and from all three non-selective transfers to non-selective solidified media plates, respectively. We analyzed 16 individual colonies each from the selective plate (plated from selective liquid transfer) and non-selective plates (plated from all non-selective liquid transfers) *via* site-specific PCR, and found that all analyzed colonies still contained the shuttle vector, independent of the presence of a selection pressure or not (**Figure S5**).

**Supplementary Text S1H – Control experiments for conjugational DNA transfer**

To investigate whether conjugational DNA transfer leads to plasmid-carrying *M. thermautotrophicus* ΔH or whether free plasmid DNA can result in genetically modified *M. thermautotrophicus* ΔH, we conducted several control experiments in addition to our standard conjugation protocol (**Figure S6,** **Materials and Methods**). First, we added 200 ng/mL of purified pMVS-V1 plasmid DNA (extracted from *E. coli* NEB stable) to a freshly inoculated non-selective *M. thermautotrophicus* ΔH culture (~1·10^5^ cells/mL). After growth of this culture to the stationary growth phase, to allow for potential natural competence to occur during all growth phases, we treated the cells similar to our standard conjugation protocol, including the non-selective recovery and selective-enrichment steps, and selective spread-plating, but without the spot-mating/plating step. Second, we substituted *E. coli* S17-1 with the non-conjugative *E. coli* NEB stable, which carried pMVS-V1, to analyze the importance of mobilization for DNA transfer into *M. thermautotrophicus*. Third, we heated *E. coli* S17-1, which carried pMVS-V1, to 60°C for 20 min *prior* to spot-mating, to check whether *E. coli* needs to be viable to mediate the DNA transfer into *M. thermautotrophicus* ΔH. Finally, and fourth, we added 250 U/mL DNase I to the *E. coli* S17-1 pre-culture during the last 30 minutes of incubation at 37°C, to reduce the amount of initial free DNA during the spot-mating process.

**Supplementary Text S1I – Quantitative β-galactosidase enzyme activity assay**

While we did not intend to perform an exhaustive promoter study here, the four selected promoters represent a subset of promoter sequences with distinctive features. The first promoter, P_synth_, is a synthetic promoter, which was based on the promoter sequence of the HmtB-encoding gene from *M. thermautotrophicus* ΔH (P*_hmtB_*) ^13^, but with several sequence modifications between the start-codon and the TATA-box sequence (**Figure 3A**) ^11^. This P_synth_ promoter was shown to be functional in *T. kodakarensis* ^11^, and we had successfully used P_synth_ to drive our neomycin-selectable marker (**Figure 1**). However, we realized after this finding that we had misinterpreted the annotation of this promoter in Santangelo, et al. ^11^, and our P_synth_ promoter did not contain the transcription factor B recognition element (BRE) sequence of archaeal promoters ^15^. Therefore, as a second promoter, we added the BRE sequence to the P_synth_ promoter sequence to generate the P_synth(BRE)_ promoter sequence, but left all modifications from Santangelo, et al. ^11^ intact (**Figure 3A**). In addition, as the third promoter, we used the promoter sequence upstream of the *hmtB*-gene from *M. thermautotrophicus* ΔH to include the native version of the promoter ^13^ on which P_synth_ and P_synth(BRE)_ were based (**Figure 3A**). Finally, as the fourth promoter, we included the promoter sequence from upstream of one of the two isoforms for the methyl-coenzyme M reductase operons from *M. thermautotrophicus* ΔH, the P*_mrt_*_(_*_M.t._*_)_ promoter (**Figure 3A**). The P*_mrt_*_(_*_M.t._*_)_ promoter does not contain the typical BRE and TATA-box sequences of archaeal promoters, but instead palindromic sequences that consist exclusively of thymine and adenine bases ^16^ (**Figure 3A**).

For a quantitative β-galactosidase enzyme activity assay, we used the chromogenic substrate ONPG (**Materials and Methods**). To implement the β-galactosidase enzyme activity assay for *M. thermautotrophicus* ΔH, we determined the β-galactosidase activity in different genetically modified *M. thermautotrophicus* ΔH strains after different incubation periods over the course of a growth experiment (**Figure S7**). Samples to assess β-galactosidase activity were taken after four different incubation periods during the growth experiment (subscript number gives time of incubation period in hours): **t_15_**, mid-exponential growth phase; **t_19_**, late-exponential growth phase; **t_23_**, early-stationary growth phase; and **t_36_**, late-stationary growth/death phase (**Figure S7**). We determined the β-galactosidase activity after these various incubation periods for four different genetically modified *M. thermautotrophicus* ΔH strains, which carry the empty vector pMVS-V1 as negative control, the pMVS1111A:P_synth_-*bgaB*, or one of two shuttle vectors, pMVS1111A:P*_hmtB_*-*bgaB* and pMVS1111A:P*_mrt_*_(_*_M.t._*_)_-*bgaB*, which have the P_synth_ promoter exchanged for the P*_hmtB_* or the P*_mrt_*_(_*_M.t._*_)_ promoter, respectively. The P_synth(BRE)_ promoter has not yet been investigated with this experiment. We found similar growth behaviors, with similar maximum OD_600_ values of ~0.4, for all four strains, and only the empty vector pMVS-V1-carrying strain reached the late-exponential growth phase already after 19 h, instead of after 23 h for the other three strains (**Figure S7A-D**). The β-galactosidase activity, given as Miller Units (**Materials and Methods**), was low, with 15±5 and 18±5 Miller Units for the empty vector control (pMVS-V1) and for the pMVS1111A:P*_mrt_*_(_*_M.t._*_)_-*bgaB*-carrying strain after all incubation periods (**Figure S7E**), respectively. For the pMVS1111A:P_synth_-*bgaB*-carrying strain, the activity increased over the course of the growth experiment to 125±15 Miller Units after 36 h of incubation (**Figure S7E**). The pMVS1111A:P*_hmtB_*_(_*_M.t._*_)_-*bgaB*-carrying strain showed the highest β-galactosidase activity with 270±20 Miller Units, while already after 19 h of incubation, no further increase in the enzyme activity was observed (**Figure S7E**).

**Supplementary Discussion**

**Supplementary Text S1J – Plating of *M. thermautotrophicus* ΔH**

In several experiments, we observed plating efficiencies of more than 100%. *Methanothermobacter* spp., including *M. thermautotrophicus* ΔH, form filaments of cells and/or show aggregation of cells depending on the growth phase and culture conditions, as has been reported before ^17,18^. This complicates cell counting in the Petroff-counting chamber and can result in an underestimation of the initial number of cells for plating, which leads to calculated plating efficiencies of more than 100%. The number of individual colonies on solidified media plates was further dependent on factors, such as the position of the plate within our anaerobic stainless-steel jar. In a typical experiment, up to ten plates were incubated in one jar, and even with plates that were prepared from the same media batch, and with the same liquid *M. thermautotrophicus* ΔH culture, we observed large variations in the number of individual colonies. Furthermore, we found considerably decreased plating efficiencies, when solidified media plates were not sufficiently dry to absorb the *M. thermautotrophicus* ΔH culture completely during the plating procedure. The accumulation of too much water can result from the formation of metabolic water during hydrogenotrophic methanogenesis ($4 H_{2}+ CO_{2} -> CH_{4}+ 2 H_{2}O$), but also due to incomplete drying of media plates after pouring hot media inside the anaerobic chamber (**Materials and Methods**). Therefore, a drying step was crucial, while this drying step should not exceed two hours in our experimental set-up, to avoid loss of carbon dioxide from the carbonate buffer system in the solidified media plates (because we used a nitrogen atmosphere in our anaerobic chamber; **Materials and Methods**). While this was sufficient as long as the humidity of the anaerobic chamber was sufficiently low, with accumulation of moisture inside the chamber, a two hour-drying step still resulted in accumulation of water in the plates. Thus, we had implemented paper clips as spacers for the petri dishes (**Figure S1E**), which helped to overcome this issue, especially when the humidity in the anaerobic chamber was high. These paper clips ensured efficient gas-solid mass transfer of molecular hydrogen and carbon dioxide, however, highest plating efficiencies were observed with sufficiently dried solidified media plates. Furthermore, the growth phase of the culture that was used for plating had a considerable effect on colony formation, especially for spread-plating (**Supplementary Text S1A**). A possible reason for this difference could be that cells are more evenly distributed during the pour-plating procedure in comparison to the spread-plating procedure, which might lead to a better accessibility for *M. thermautotrophicus* ΔH to media components, or more stable pH conditions.

**Supplementary References**

1 Sarmiento, F., Leigh, J. A. & Whitman, W. B. Genetic systems for hydrogenotrophic methanogens. *Methods Enzymol* **494**, 43-73 (2011).

2 Buan, N., Kulkarni, G. & Metcalf, W. Genetic methods for *Methanosarcina* species. *Methods in enzymology* **494**, 23-42 (2011).

3 Susanti, D., Frazier, M. C. & Mukhopadhyay, B. A genetic system for *Methanocaldococcus jannaschii:* An evolutionary deeply rooted hyperthermophilic methanarchaeon. *Front Microbiol* **10**, 1256 (2019).

4 Enzmann, F., Mayer, F., Rother, M. & Holtmann, D. Methanogens: Biochemical background and biotechnological applications. *AMB Express* **8**, 1 (2018).

5 Heap, J. T., Pennington, O. J., Cartman, S. T. & Minton, N. P. A modular system for *Clostridium* shuttle plasmids. *J Microbiol Methods* **78**, 79-85 (2009).

6 Bokranz, M., Klein, A. & Meile, L. Complete nucleotide sequence of plasmid pME2001 of *Methanobacterium thermoautotrophicum* (Marburg). *Nucleic Acids Res* **18**, 363 (1990).

7 Luo, Y., Leisinger, T. & Wasserfallen, A. Comparative sequence analysis of plasmids pME2001 and pME2200 of *Methanothermobacter marburgensis* strains Marburg and ZH3. *Plasmid* **45**, 18-30 (2001).

8 Meile, L. & Reeve, J. N. Potential shuttle vectors based on the methanogen plasmid pME2001. *Nature Biotechnol* **3**, 69-72 (1985).

9 Meile, L., Madon, J. & Leisinger, T. Identification of a transcript and its promoter region on the archaebacterial plasmid pME2001. *J Bacteriol* **170**, 478-481 (1988).

10 Shaw, A. J., Hogsett, D. A. & Lynd, L. R. Natural competence in *Thermoanaerobacter* and *Thermoanaerobacterium* species. *Appl Environ Microbiol* **76**, 4713-4719 (2010).

11 Santangelo, T. J. *et al.* Polarity in archaeal operon transcription in *Thermococcus kodakaraensis*. *J Bacteriol* **190**, 2244-2248 (2008).

12 Gernhardt, P., Possot, O., Foglino, M., Sibold, L. & Klein, A. Construction of an integration vector for use in the archaebacterium *Methanococcus voltae* and expression of a eubacterial resistance gene. *Mol Gen Genet* **221**, 273-279 (1990).

13 Tabassum, R., Sandman, K. & Reeve, J. HMt, a histone-related protein from *Methanobacterium thermoautotrophicum* ΔH. *J Bacteriol* **174**, 7890-7895 (1992).

14 Darcy, T. J. *et al.* *Methanobacterium thermoautotrophicum* RNA polymerase and transcription *in vitro*. *J Bacteriol* **181**, 4424-4429 (1999).

15 Allers, T. & Mevarech, M. Archaeal genetics - the third way. *Nat Rev Genet* **6**, 58-73 (2005).

16 Shinzato, N. *et al.* Specific DNA binding of a potential transcriptional regulator, inosine 5'-monophosphate dehydrogenase-related protein VII, to the promoter region of a methyl coenzyme M reductase I-encoding operon retrieved from *Methanothermobacter thermautotrophicus* strain ΔH. *Appl Environ Microbiol* **74**, 6239-6247 (2008).

17 Majernik, A. I., Lundgren, M., McDermott, P., Bernander, R. & Chong, J. P. DNA content and nucleoid distribution in *Methanothermobacter thermautotrophicus*. *J Bacteriol* **187**, 1856-1858 (2005).

18 Kiener, A. & Leisinger, T. Oxygen sensitivity of methanogenic bacteria. *Syst Appl Microbiol* **4**, 305-312 (1983).
