# Supplementary figures and images for: A Shuttle-Vector System Allows Heterologous Gene Expression in the Thermophilic Methanogen Methanothermobacter thermautotrophicus ΔH
Source: mBio. 2021 Nov 23;12(6):e02766-21. doi: 10.1128/mBio.02766-21 (PMC8609365; doi:10.1128/mBio.02766-21)

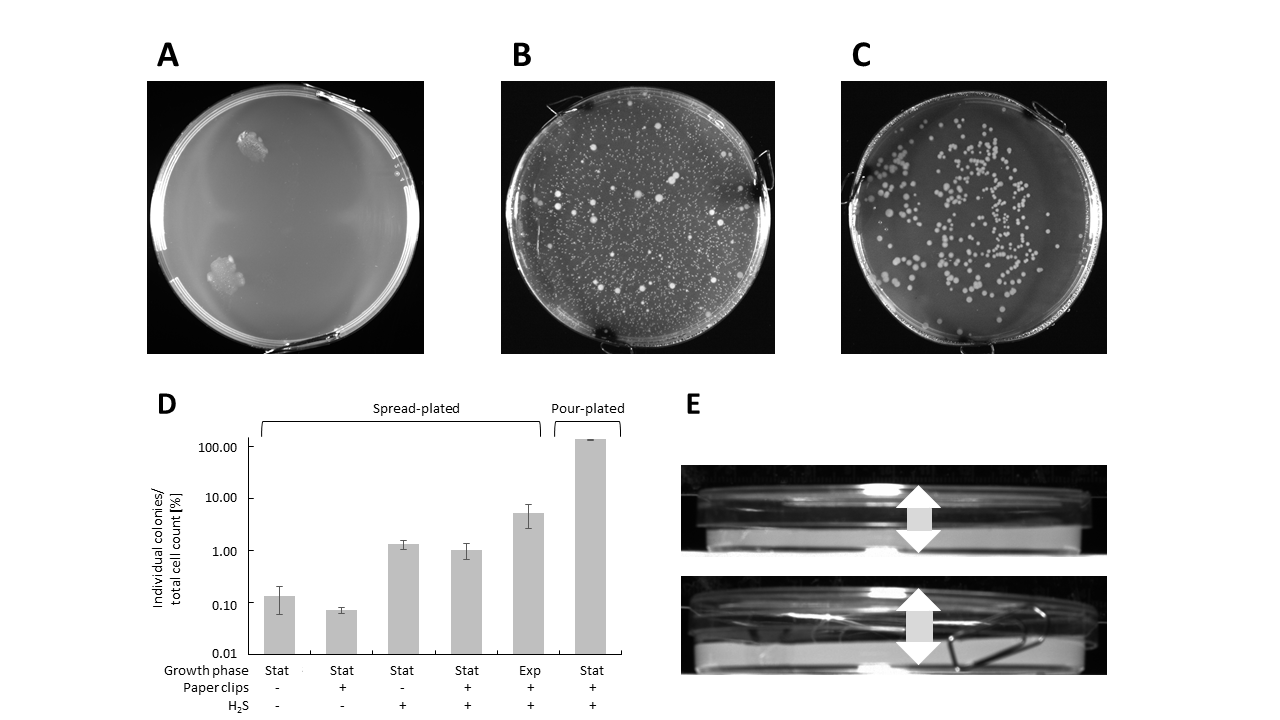

Supplement: FIG S1 [file mbio.02766-21-sf001.tif]

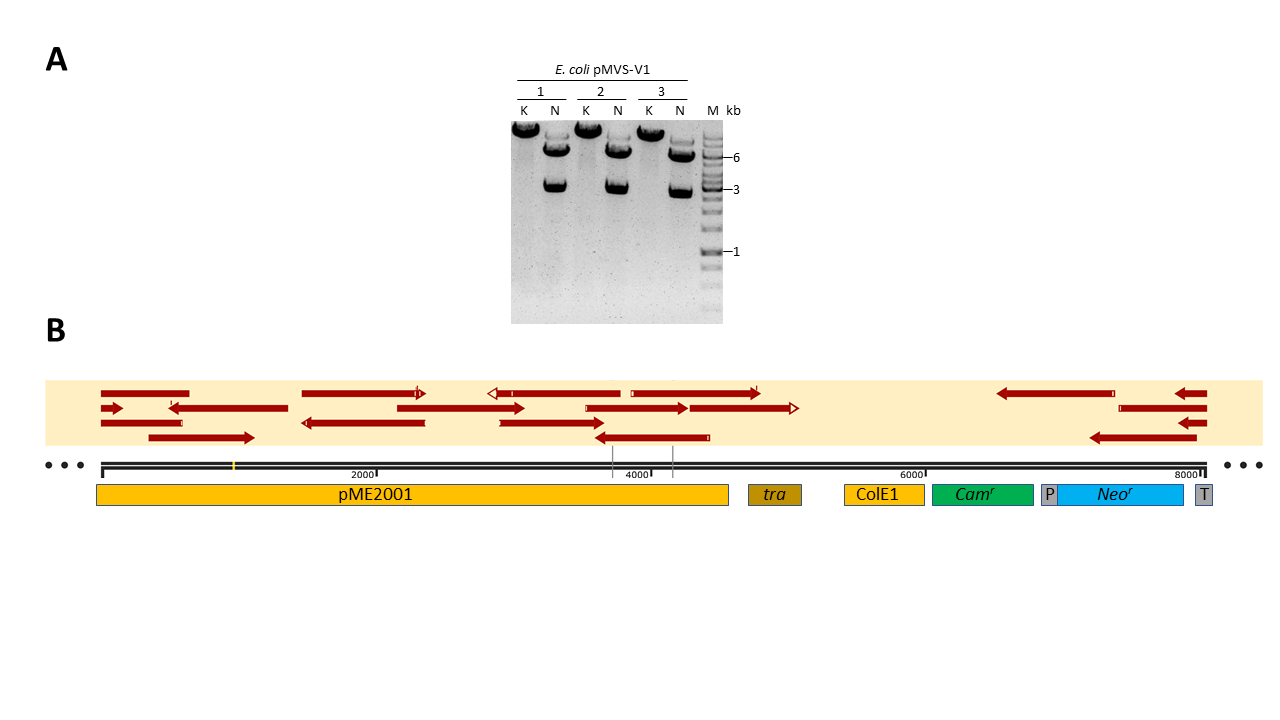

Supplement: FIG S4 [file mbio.02766-21-sf004.tif]

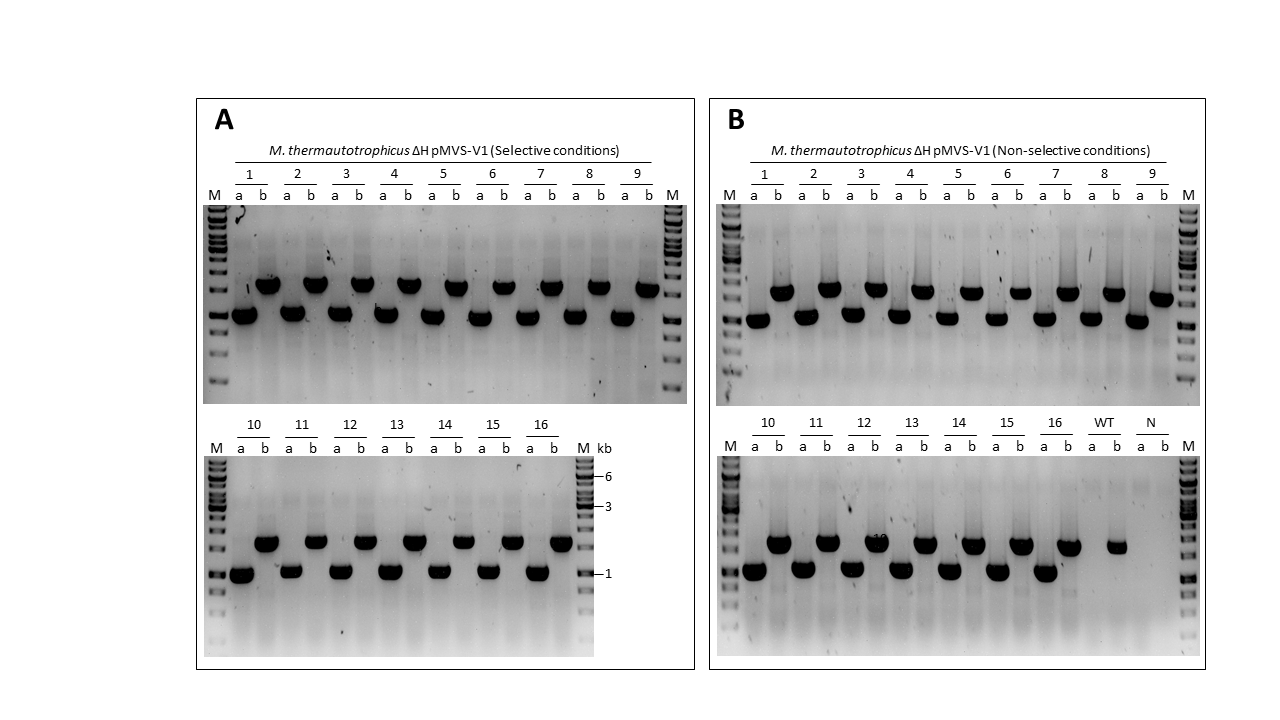

Supplement: FIG S5 [file mbio.02766-21-sf005.tif]

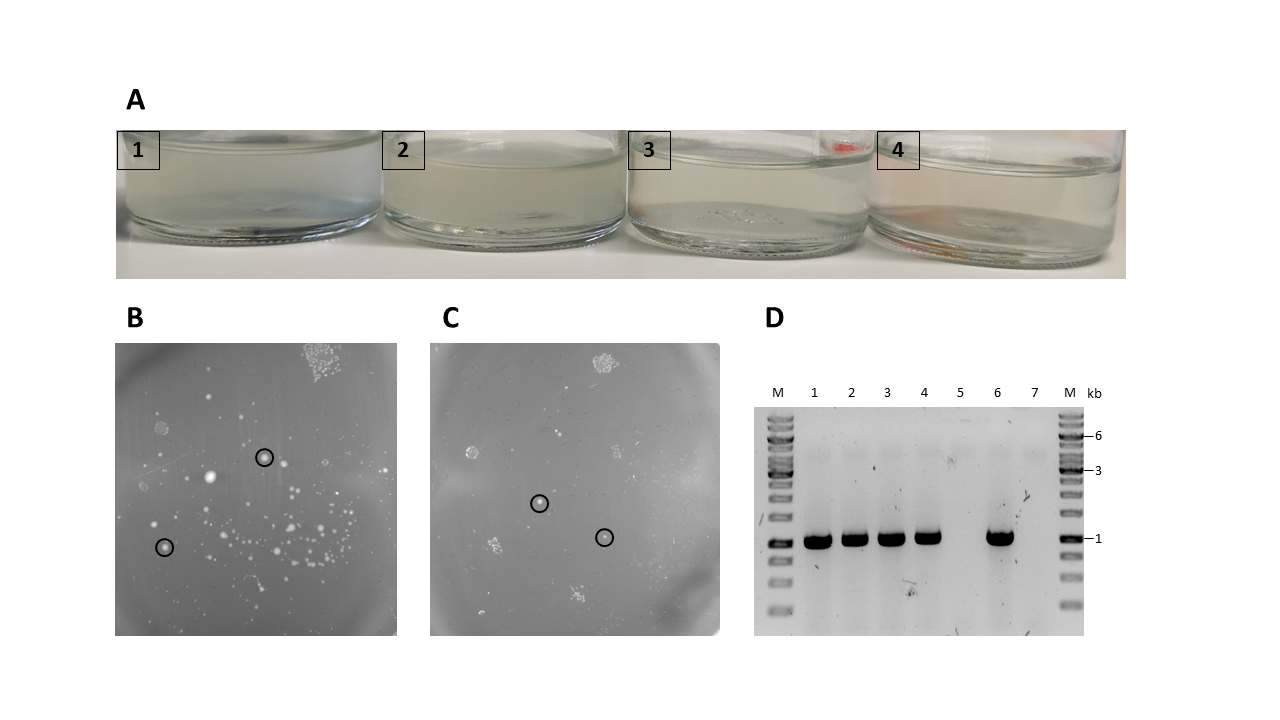

Supplement: FIG S6 [file mbio.02766-21-sf006.tif]
