# Supplementary material for: A Shuttle-Vector System Allows Heterologous Gene Expression in the Thermophilic Methanogen Methanothermobacter thermautotrophicus ΔH
Source: mBio. 2021 Nov 23;12(6):e02766-21. doi: 10.1128/mBio.02766-21 (PMC8609365; doi:10.1128/mBio.02766-21)
Supplement: TABLE S1 [file mbio.02766-21-st001.docx]

**Table S1.** Example calculations for conjugation frequencies for DNA transfer into *M. thermautotrophicus* ΔH based on Equation S1

| Parameters | N_P_ | E | V_R_ | D_R_ | N_0_ | R_W_ | D_S_ | Conjugation frequency |
| --- | --- | --- | --- | --- | --- | --- | --- | --- |
| Best case scenario | 10 | 0.01 | 50 | 5 | 5·10^8^ | 0.5 | 0 | 6·10^-6^ |
| Worst case scenario | 1 | 0.05 | 50 | 8 | 5·10^8^ | 1 | 1 | 4·10^-9^ |
| Most likely scenario | 5 | 0.02 | 50 | 6 | 5·10^8^ | 0.8 | 0 | 5·10^-7^ |
